# SPRTN protease and checkpoint kinase 1 cross-activation loop safeguards DNA replication

**DOI:** 10.1038/s41467-019-11095-y

**Published:** 2019-07-17

**Authors:** Swagata Halder, Ignacio Torrecilla, Martin D. Burkhalter, Marta Popović, John Fielden, Bruno Vaz, Judith Oehler, Domenic Pilger, Davor Lessel, Katherine Wiseman, Abhay Narayan Singh, Iolanda Vendrell, Roman Fischer, Melanie Philipp, Kristijan Ramadan

**Affiliations:** 1 Cancer Research UK and Medical Research Council Oxford Institute for Radiation Oncology, Department of Oncology, University of Oxford, Roosevelt Drive, Oxford, OX3 7DQ UK; 2 Institute of Biochemistry and Molecular Biology, Ulm University, Albert-Einstein-Allee 11, 89081 Ulm, Germany; 3 Department of Experimental and Clinical Pharmacology and Pharmacogenomics, University of Tübingen, 72074 Tübingen, Germany; 4 Institute Ruder Boškovic, Bijenička Cesta 54, 10000 Zagreb, Croatia; 5 Institute of Human Genetics, University Medical Center Hamburg-Eppendorf, 20246 Hamburg, Germany; 6 TDI Mass Spectrometry Laboratory, Target Discovery Institute, Nuffield Department of Medicine, University of Oxford, Oxford, OX3 7FZ UK

**Keywords:** Replisome, Proteolysis

## Abstract

The SPRTN metalloprotease is essential for DNA-protein crosslink (DPC) repair and DNA replication in vertebrate cells. Cells deficient in SPRTN protease exhibit DPC-induced replication stress and genome instability, manifesting as premature ageing and liver cancer. Here, we provide a body of evidence suggesting that SPRTN activates the ATR-CHK1 phosphorylation signalling cascade during physiological DNA replication by proteolysis-dependent eviction of CHK1 from replicative chromatin. During this process, SPRTN proteolyses the C-terminal/inhibitory part of CHK1, liberating N-terminal CHK1 kinase active fragments. Simultaneously, CHK1 full length and its N-terminal fragments phosphorylate SPRTN at the C-terminal regulatory domain, which stimulates SPRTN recruitment to chromatin to promote unperturbed DNA replication fork progression and DPC repair. Our data suggest that a SPRTN-CHK1 cross-activation loop plays a part in DNA replication and protection from DNA replication stress. Finally, our results with purified components of this pathway further support the proposed model of a SPRTN-CHK1 cross-activation loop.

## Abstract (web summary)

Cells deficient in SPRTN protease activity exhibit severe DNA-protein crosslink induced replication stress and genome instability. Here the author reveal a functional link between the SPRTN protease and the CHK1 kinase during physiological DNA replication.

## Introduction

The timely completion of DNA replication is essential for genome integrity and preventing the onset of cancer, premature ageing and developmental disorders^1,2^. DNA replication is constantly threatened by many factors, including DNA lesions, collisions with the transcription machinery and repetitive DNA sequences. Cells have evolved robust DNA damage tolerance and DNA damage response pathways to cope with the different lesions and obstacles that challenge the progression of DNA replication forks^3–6^. In particular, the ataxia telangiectasia and Rad3 related (ATR)-CHK1 signalling cascade is the major regulator of the response to replication stress^7,8^. This cascade performs multiple functions in response to DNA replication stress, including regulating DNA replication origin firing, stabilising stalled replication forks and delaying mitotic entry by preventing cyclin-dependent kinase (CDK) 1 and 2 hyper-activation^5,9^.

The main stimulus for ATR-CHK1 activation is replication protein A (RPA)-coated single-stranded (ss) DNA that typically forms upon DNA replication fork stalling due to uncoupling of the cell division cycle 45 (Cdc45), minichromosome maintenance protein complex 2-7 (Mcm2-7) and go-ichi-ni-san protein complex GINS (CMG) helicase complex from DNA polymerases^5^. This ssDNA-protein structure recruits the ATR–ATR-interacting protein (ATRIP) kinase complex which, with the help of Ewing’s tumour-associated antigen 1 (ETAA1) and DNA topoisomerase 2-binding protein 1 (TopBP1), activates CHK1 by phosphorylating serines 317 and 345 to expose the catalytic N-terminal domain of CHK1^10,11^. Upon phosphorylation, CHK1 is released from chromatin by an as yet unknown mechanism and spread throughout the nucleus and cytoplasm to regulate the activity of its substrates and safeguard genome stability^9,12,13^. The ATR-CHK1 signalling pathway can also be activated in S-phase by ssDNA generated after 5′-3′ end resection of double strand DNA breaks (DSB)^14^.

However, given that ATR-CHK1 kinase activity is required for physiological DNA replication fork progression—when long stretches of ssDNA are scarce and the robust activation of ATR-CHK1 signalling cascade would be deleterious for cells—the question of how ATR-CHK1 is activated during steady-state DNA synthesis remains unanswered^15–19^. Identifying the mechanisms that regulate ATR-CHK1 signalling under physiological conditions is therefore essential to understand how cells survive and preserve genomic stability^20^. Recent work identified ETAA1 as an activator of ATR during steady-state DNA replication^11,21–24^. However, this finding does not explain yet how CHK1, as a main signalling component downstream of ATR, is released from chromatin to activate the ATR-CHK1-CDC25-CDK1/2 signalling cascade.

We and others have recently identified the SPRTN metalloprotease as being a constitutive component of the DNA replication machinery and necessary for DNA replication^25–30^. The essential role of SPRTN in safeguarding genome stability is demonstrated both in human disease and in animal models. Monogenic, biallelic *SPRTN* germline mutations cause Ruijs–Aalfs Syndrome (RJALS), a rare disease characterised by genomic instability, premature ageing and hepatocellular carcinoma^25,31,32^. Furthermore, SPRTN hypomorphic mice develop RJALS-like phenotypes, while complete SPRTN knockout is embryonic lethal^33^. SPRTN has recently been identified as an essential core fitness gene in humans^34–36^. Finally, downregulation of SPRTN in zebrafish severely impairs normal embryonic development and increases embryonic lethality^31^.

The source of genome instability in RJALS patient derived cells and in SPRTN-deficient human and mouse cells was recently demonstrated to arise from replication stress caused by the accumulation of replication-blocking DNA-protein crosslinks (DPC)^25,27,28^. DPCs are formed by various aldehydes including formaldehyde (FA), formed as metabolic by-products of lipid peroxidation and histone and DNA demethylation^37–39^. As SPRTN protease activity is required to cleave DPCs, defective SPRTN protease activity results in profound replication stress, visualised as increased fork stalling and significantly reduced DNA replication fork velocity. Strikingly, we observed a severe G2/M-checkpoint defect in SPRTN-deficient cells treated with genotoxic agents that interfere with DNA replication^31^. The G2/M checkpoint was, however, completely functional after the induction of non-replication-associated DNA strand breaks using ionising radiation, suggesting that SPRTN-defective cells lack the ability to activate CHK1 in response to replication stress when replication forks are still intact^31^.

Our results here suggest that SPRTN protease stimulates CHK1 kinase function during physiological DNA replication and vice versa. Specifically, SPRTN cleaves, evicts and activates CHK1 from replicative chromatin, enabling physiological CHK1 function during steady-state DNA replication. Our data suggests that SPRTN proteolysis can cleave the regulatory/inhibitory C-terminal domain of CHK1 in vitro and in vivo, and the released N-terminal CHK1 products are kinase active. These N-terminal CHK1 products, when ectopically expressed, are sufficient to stabilise DNA replication forks in a CHK1 kinase-dependent but ATR-independent manner. The N-terminal CHK1 fragments also rescue embryonic development defect and genome instability in cells where SPRTN is depleted to approximately 30% of wildtype cells. This rescue is dependent of CHK1 phosphorylating the C-terminus of the residual SPRTN, further promoting SPRTN recruitment to chromatin for the removal of DPCs in front of unperturbed DNA replication fork progression. Finally, activation of CHK1 by SPRTN proteolysis in vitro further supports our proposed model of a SPRTN-CHK1 cross-activation loop.

## Results

### SPRTN deficiency leads to aberrant ATR-CHK1 signalling cascade

**Fig. 1:**
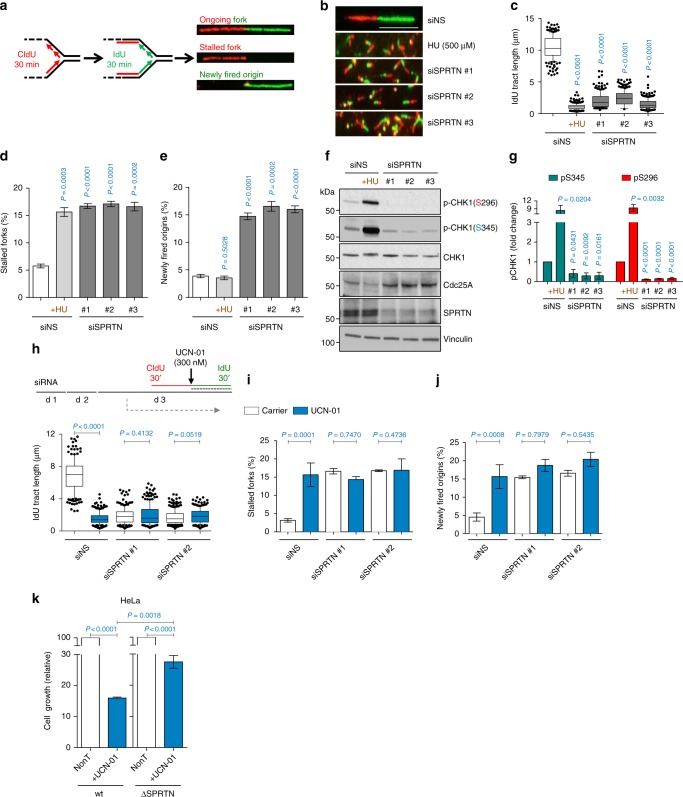
SPRTN depleted cells endure severe DNA replication stress but fail to activate a CHK1 response. **a** Schematic representation of DNA fiber assay. See also Methods. **b** DNA fibers obtained from HEK293 cells that have been treated with the indicated siRNAs against SPRTN (siSPRTN) or with hydroxyurea (HU). Scale bar: 10 µm. Data shown are representative images of three independent experiments. **c**–**e** DNA fiber assay analysis showing replication fork length (**c**), stalled replication forks (**d**) and newly fired origins (**e**). **c** siSPRTN cells exhibit decreased fork velocity. >100 individual IdU tracts were measured per experiment per condition. Data are shown as mean with 25–75% percentile range (box) and 10–90% percentile (whiskers); *n* = 3 independent experiments, two-tailed Student's *t*-test. **d** siSPRTN cells exhibit an increased frequency of stalled replication forks. >400 forks were scored per condition per experiment. Mean ± SEM; *n* = 3 independent experiments, two-tailed Student's *t*-test. **e** siSPRTN cells exhibit increased newly firing of dormant origin, contrary to cells treated with HU. >400 forks were scored per condition per experiment. Mean ± SEM; *n* = 3 independent experiments, two-tailed Student's *t*-test. **f** Knock-down of SPRTN in HEK293 cells diminishes the phosphorylation status of CHK1 residues S296 and S345, and enhances Cdc25A stability, contrary to treatment with HU. Immunoblots were run with whole cell lysates and represent three independent experiments. **g** Quantifications for (**f**) of CHK1 phosphorylation signal at residues S345 and S296, normalised to vinculin. Mean ± SEM; *n* = 3 independent experiments, two-tailed Student's *t*-test. **h**–**j** Treatment of cells with the CHK1 inhibitor UCN-01 induces severe replication stress to a similar extent as SPRTN-inactivation, detected by DNA fiber assay analysis. Schematic representation of experimental layout showing the addition of UCN-01 with IdU is also shown in (**h**, top); d: day. **k** Cell growth of *wt* and SPRTN-deficient (ΔSPRTN) HeLa cells in response to the CHK1 inhibitor UCN-01 (300 nM) on day 4 after seeding cells at same density (day 0). Mean ± SEM; *n* = 3 independent experiments, two-tailed Student's *t*-test. Source data for (**c**–**k**) are provided as a Source Data file

Analysis of RJALS patient and SPRTN-depleted cells revealed that SPRTN protease activity is essential for DNA replication fork progression, cell cycle progression and G2/M checkpoint activation after DNA replication stress but not ionising radiation^25,28,31^. These results suggest that SPRTN bridges DNA replication and G2/M-checkpoint regulation. To re-evaluate these findings, we performed analysis of DNA replication using the DNA fiber assay (Fig. 1a). Depletion of SPRTN by three independent siRNA sequences caused severe DNA replication stress in human embryonic kidney 293 (HEK293) cells, visualised as a reduction in DNA replication fork velocity and an increased frequency of fork stalling (Fig. 1b–d). Short treatment of control cells with a low dose of hydroxyurea (HU), a drug that limits the cellular dNTP pool, was used as a positive control of DNA replication stress phenotypes. In general, as a response to DNA replication stress, cells suppress dormant origin firing, as was visible after HU treatment (Fig. 1e). Interestingly, dormant origin firing was more than 3-fold higher in SPRTN-depleted cells when compared to HU-treated cells. As firing of dormant origins is tightly regulated by the CHK1 kinase^40,41^, which also controls the G2/M-checkpoint^42,43^, we asked whether the ATR-CHK1 signalling was defective in SPRTN-inactivated cells. ATR-CHK1 signalling, visualised by CHK1 S345 phosphorylation, was not activated in SPRTN-depleted cells (Fig. 1f, g) despite these cells showing the classical signatures of DNA replication stress visualised by the DNA fibers (Fig. 1b–e). Accordingly, CHK1 kinase activity was not activated as demonstrated by the lack of CHK1 phosphorylation on serine 296 (S296), the residue that CHK1 auto-phosphorylates once its kinase activity has been stimulated by ATR^44,45^. Furthermore, despite being in similar cell cycle stages (Supplementary Fig. 1a–c), SPRTN-depleted cells exhibited less CHK1-S296 and -S345 phosphorylation than even unchallenged control cells and ∼9–10-fold less than HU-treated control cells, which showed similarly reduced replication fork velocity and elevated levels of fork stalling as SPRTN-depleted cells (Fig. 1f, g). Similar findings were observed in HeLa cells (see below). These results show that SPRTN-inactivated human cells fail to activate a robust ATR-CHK1 response despite exhibiting severe replication stress phenotypes that would ordinarily be expected to elicit such a response, namely CHK1 chromatin eviction and consequent activation of CHK1 signalling^12^.

Indeed, SPRTN-inactivated cells accumulate over 2-fold more CHK1 on chromatin when compared to unchallenged control cells, a similar effect as in cells treated with the DNA-protein crosslinking agent formaldehyde (FA), but opposite to cells treated with HU, where CHK1 is released from chromatin (Supplementary Fig. 1d, e).

To further validate this observation, we treated HEK293 cells with UCN-01, a well-characterised CHK1 inhibitor^16,46,47^. UCN-01 treatment in control cells caused a severe reduction in replication fork velocity and increased the frequency of new (dormant) origin firing and fork stalling (Fig. 1h–j). UCN-01, however, had no additive effect on replication fork velocity, new origin firing, or fork stalling in SPRTN-depleted cells. In addition, in comparison to wt cells, SPRTN-haploinsufficient HeLa (ΔSPRTN) cells were relatively much less sensitive to UCN-01 treatment (Fig. 1k). This further suggests that CHK1 is not fully activated in SPRTN-defective human cells. Altogether, these results highlight the importance of CHK1 activity during steady-state DNA replication^15,16,19^, demonstrate an epistatic relationship between SPRTN and CHK1, and reveal a severe defect in CHK1 kinase activation in SPRTN-defective cells despite them suffering from severe DNA replication stress. We concluded that SPRTN-defective cells lack the optimal (physiological) ATR-CHK1 activity required for DNA replication and genome stability, due to their inefficiency in evicting CHK1 from chromatin, and thus activating a steady-state CHK1 signalling cascade.

### SPRTN regulates CHK1 activity under physiological conditions

To test this conclusion, we investigated the signalling pathway downstream of ATR-CHK1 by monitoring the total levels of the CHK1 target, protein phosphatase Cdc25A^3^. Upon phosphorylation by CHK1, Cdc25A is degraded by the proteasome, as observed in cells treated with HU (Fig. 1f and Supplementary Fig. 1f). Consequently, CDK1/2 are hyper-phosphorylated and become inactive, which leads to intra S-phase and G2/M-checkpoint activation and cell cycle arrest^48,49^. CDK1 and 2 drive S-phase progression and the G2/M cell cycle transition, but hyper-activation of CDK1/2 negatively influences DNA replication fork stability and causes premature mitotic entry^50,51^. Hence, the ATR-CHK1-Cdc25-CDK1/2 pathway is necessary to regulate cell cycle progression during DNA synthesis. Due to faulty CHK1 activation, SPRTN-depleted cells hyper-accumulated Cdc25A (Fig. 1f and Supplementary Fig. 1f), which in turn dephosphorylates and hyper-activates CDK1/2, as was visible by the increased phosphorylation of total CDK1/2 substrates in HEK293 cell extracts (Supplementary Fig. 1g, h).

### CHK1 overexpression corrects SPRTN-defective phenotypes

**Fig. 2:**
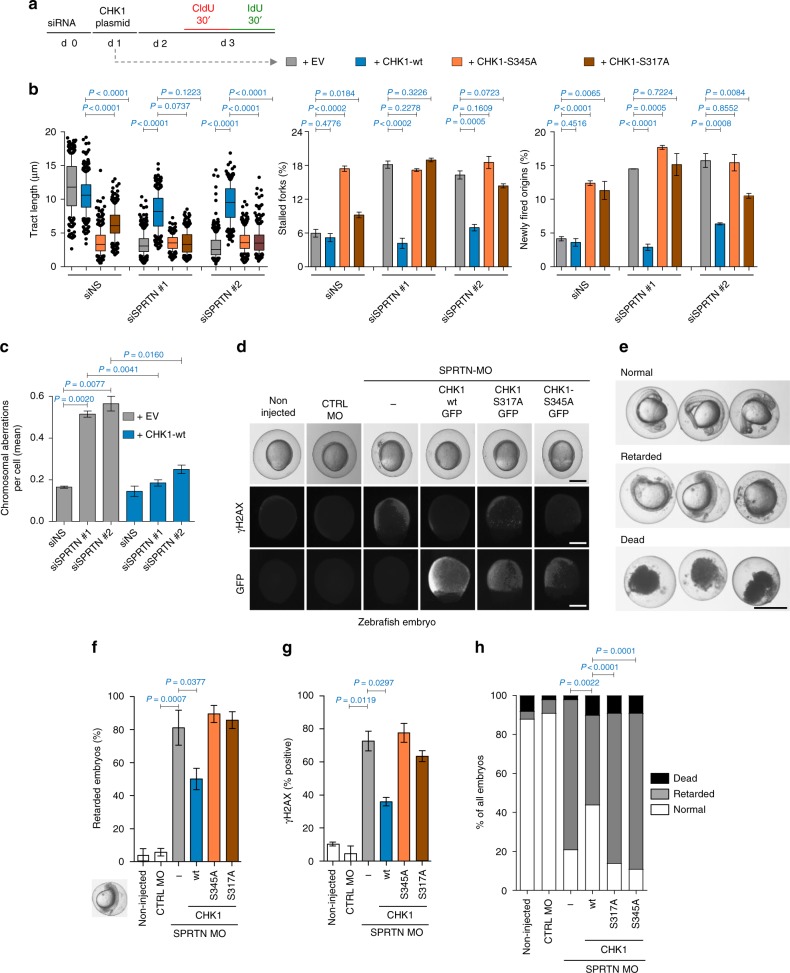
Ectopic CHK1 overexpression rescues SPRTN-deficiency phenotypes. **a** Experimental strategy followed to assess the effect of different CHK1 variants on DNA replication by DNA fiber assay (d: day, EV: empty vector). **b** Rescue of DNA replication defects in SPRTN-depleted HEK293 cells with ectopic expression of CHK1-wt, but not with the phospho-deficient CHK1 variants. Graphs show quantification data from DNA fibers for replication fork velocity (left panel; mean ± 25–75 percentile range (box) ± 10–90 percentile range (whiskers)), newly fired origins (centre panel; mean ± SEM) and stalled replication forks (right panel; mean ± SEM); >100 DNA fibers were analysed per condition and experiment; *n* = 3 independent replicates, two-tailed Student's *t*-test. **c** Overexpression of CHK1-wt diminishes the number of chromosomal aberrations caused by SPRTN knock-down visualised by metaphase spreads from HEK293 cells. >30 cells were scored per condition per experiments; mean ± SEM; *n* = 3 individual experiments, two-tailed Student's *t*-test. **d** Representative images of zebrafish embryos non-injected or injected with either a control or a previously characterised morpholino (MO) against SPRTN at 9–10 h post fertilization (hpf). To assess CHK1 function, capped RNA encoding different mutants of GFP-tagged human CHK1 were co-injected with the SPRTN MO. Upper row: live images. Middle row: pictures of γH2AX to show DNA damage. Lower row: Pictures of GFP to show expression of CHK1 protein variants. **e** Representative images for normal, retarded and dead zebrafish embryos at 24 hpf. Scale bar = 500 µm. **f**–**h** Overexpression of CHK1-wt, but not of the phospho-deficient CHK1 variants, rescues developmental defects and DNA damage induced by SPRTN depletion in zebrafish embryos. Percentage of zebrafish embryos that show developmental retardation relative to living embryo are represented in (**f**) (mean ± SEM; >70 embryo were scored in *n* = 4 independent experiments, two-tailed Student's *t*-test), whereas data scoring normal, retarded and dead zebrafish embryos are represented in (**h**), Fisher’s exact test. γH2AX, marker of DNA damage, is represented in (**g**) (mean ± SEM; at least 30 GFP-positive embryos were counted per condition; *n* = 2 replicates, two-tailed Student's *t*-test). Source data for (**b**, **c**, **f**–**h**) are provided as a Source Data file

To assess whether the failure to activate ATR-CHK1 signalling could explain the DNA replication phenotypes and G2/M defects observed in SPRTN-depleted cells (Fig. 1)^31^, we ectopically expressed CHK1-wild type (wt) or its ATR-dependent phosphorylation (phospho)-defective variants (CHK1-S317A or CHK1-S345A) (Fig. 2a, b and Supplementary Fig. 2a). CHK1-wt, but not CHK1-S317A or CHK1-S345A, restored DNA replication fork velocity, suppressed new origin firing and rescued replication fork stalling in SPRTN-depleted cells. Moreover, ectopic expression of CHK1-wt in SPRTN-deficient cells also corrected chromosomal instability, measured by the number of chromosomal aberrations on mitotic chromosomes (Fig. 2c and Supplementary Fig. 2b). These results further support our initial observation that SPRTN-inactivation leads to an impaired ATR-CHK1 signalling cascade, resulting in severe DNA replication stress, a defective G2/M checkpoint and the accumulation of chromosomal aberrations in SPRTN-deficient cells^31,33^. These results also suggest that the ATR kinase is required for the full CHK1 activation in vivo as the CHK1 variants, S317A and S345A (ATR-phosphorylation sites), are dominant negative even in wild type cells (Fig. 2a, b) where SPRTN function is intact. These results are in consonance with previously published literature on CHK1^44,52^.

### The SPRTN-ATR-CHK1 axis is essential in zebrafish embryos

To investigate and validate our observations so far on an organismal level, we took advantage of the zebrafish model system. We have previously shown that morpholino (MO)-mediated depletion of SPRTN in zebrafish embryos causes severe development defects and accumulation of DNA damage^31^. When fertilized eggs with SPRTN MO were co-injected with capped RNAs encoding for GFP-CHK1-wt, both developmental retardation and DNA damage (the latter analysed by γH2AX accumulation) were rescued (Fig. 2d–h and Supplementary Fig. 2c). Conversely, reconstitution with RNAs encoding ATR-dependent phospho-defective variants of GFP-CHK1, S317A or S345A, failed to rescue the phenotypes of SPRTN depletion in early zebrafish embryos. Altogether, these data suggest that the restoration of CHK1 activity is able to compensate for SPRTN-deficiency in human cells and zebrafish embryos, and that the SPRTN-CHK1 axis is conserved in vertebrates and dependent on ATR activity.

### SPRTN does not contribute to CHK1 activation after DNA breaks

**Fig. 3:**
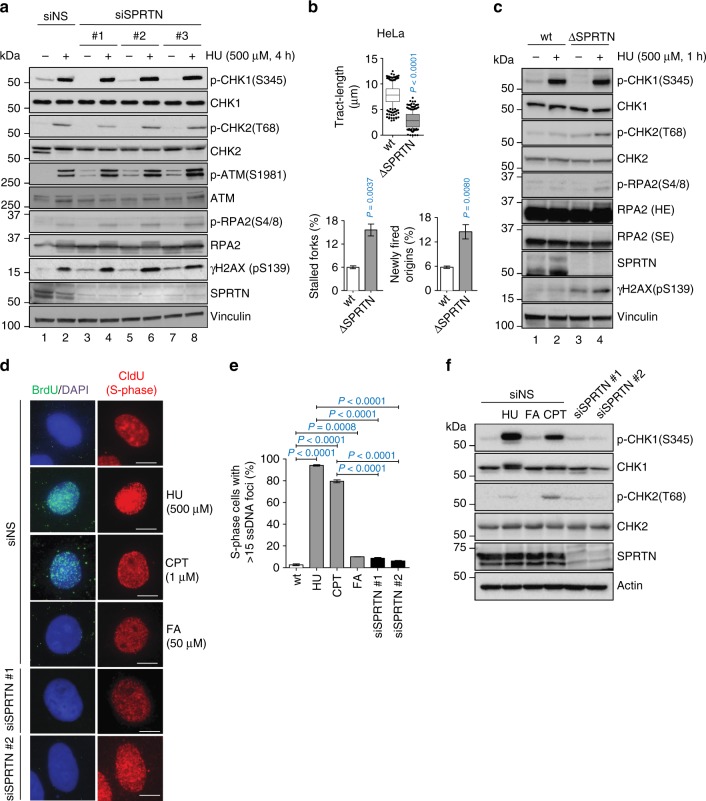
SPRTN does not contribute to the activation of CHK1 after DSB formation. **a** Phosphorylation status of the ATR-CHK1 and ATM-CHK2 pathways in control (siNS) and siRNA SPRTN-depleted HEK293 cells under unchallenged condition or when cells were treated with hydroxyurea (HU). Whole cell extracts were used for the immunoblots. **b** DNA fiber assay analysis comparing HeLa-wt (*wild type*, parental) and HeLa-ΔSPRTN cells: quantification of replication fork velocity (top; mean ± 25–75 percentile range (box) ± 10–90 percentile range (whiskers)), newly fired origins (bottom left; mean ± SEM) and stalled replication forks (bottom right; mean ± SEM). >100 DNA fibers were analysed per condition and experiment; *n* = 3 experimental replicates, two-tailed Student's *t*-test. **c** Analysis of the phosphorylation status of the ATR-CHK1 and ATM-CHK2 pathways comparing HeLa -*wt* and -ΔSPRTN under unchallenged or HU-treated conditions. Whole cell extracts were used for the immunoblots. SE: short exposure; LE: long exposure. **d**, **e** SPRTN-deficient (siSPRTN) or wt cells (siNS) treated with formaldehyde (FA; 50 μM, 1 h) do not exhibit robust single-stranded DNA foci in S-phase cells (CldU positive). HU (500 μM, 1 h) was used as a positive control to generate replication stress-induced ssDNA formation. Camptothecin (CPT, 1 μM, 1 h followed by 1 h recovery) was used as a positive control for ssDNA formation induced by DSBs. Data are shown as representative immunofluorescent microscopy images of BrdU foci (ssDNA) in S-phase (CldU positive) HeLa cells (**d**), and as quantification of cells with more than 15 BrdU foci (**e**). Scale bar = 10 µm. >70 individual CldU positive cells were scored per condition per experiment. Mean ± SEM; *n* = 3 experimental replicates, two-tailed Student's *t*-test. **f** FA treatment and SPRTN-depletion fail to induce phosphorylation of CHK1 at Ser345 due to the absence of ssDNA formation. HU or CPT were used at the same conditions as in (**d**) as positive controls for ssDNA-induced CHK1 and CHK2 activation. Immunoblots of HeLa cells whole cell extracts shown here represent three experimental replicates. Source data for (**a**–**c**, **e**, **f**) are provided as a Source Data file

Interestingly, when SPRTN-depleted cells were challenged with HU, CHK1 was activated to the same extent as in HU-treated control cells (Fig. 3a, lanes 2, 4, 6 and 8). This CHK1 activation was concomitant with phosphorylation of ATM, CHK2 and RPA, indicating DSB formation^42,53^. Moreover, the total level of RPA remained unchanged in these cells and phosphorylation of RPA ruled out the possibility that severe replication stress in SPRTN-inactivated cells results from RPA exhaustion^54^. Similar findings were observed in haploinsufficient HeLa SPRTN cells (Δ-SPRTN) (Fig. 3b, c). Taken together, our data suggest that SPRTN-deficient human cells have a fully functional ATR-CHK1 signalling pathway in the context of DSB repair, but that SPRTN is needed to activate this pathway during physiological/steady-state DNA replication.

In addition, immunofluorescence-coupled microscopy analysis of ssDNA formation in S-phase cells (CldU positive) by BrdU staining under native conditions further suggested that SPRTN-defective cells do not form extensive ssDNA (Fig. 3d–f), a platform for canonical and robust ATR-CHK1 activation. Similar results were obtained with FA-treatment. HU or Camptothecin (CPT) treatment was used as a positive control for DNA replication stress-induced ssDNA formation. However, when SPRTN-depleted cells were exposed to a high dose of CPT or HU, a striking increase in ssDNA (visualised as BrdU foci), generated by 5′-3′ end resection of DSBs (Supplementary Fig. 3a, b), was observed. This effect ruled out the possibility that this BrdU/ssDNA assay was compromised in SPRTN-deficient cells owing to reduced BrdU incorporation due to defective DNA replication (Supplementary Fig. 1a–c). Altogether, these results suggest that SPRTN plays a role in the activation of a physiological ATR-CHK1 response during steady-state DNA synthesis but is dispensable for CHK1 activation after DSB formation, when substantial amounts of ssDNA is formed by 5′-3′ end resection.

### SPRTN proteolysis releases CHK1 from replicating chromatin

Given that SPRTN enables DNA replication by proteolysis of its chromatin-bound substrates^25,27^ and that, in SPRTN-defective cells, the ATR-CHK1 signalling cascade is not activated during unperturbed conditions (Fig. 1), we considered potential interactions between SPRTN and ATR-CHK1. First, we tested if SPRTN forms a physical complex with ATR and CHK1 by pull-down assays with SPRTN-wt from HEK293 cells under normal conditions or during DNA replication stress induced by a low dose of CPT (100 nM, Supplementary Fig. 4a)^55^. We verified that components of DNA replication such as PCNA co-precipitate with SPRTN along with CHK1. Interestingly, ATR could not be detected in a SPRTN complex here. As CHK1, but not ATR, form a physical complex with SPRTN and since the mechanism of CHK1 release from chromatin to ensure proper activation of the CHK1 signalling cascade is not known, we hypothesised that SPRTN may directly regulate CHK1 and thus contribute to the ATR-CHK1 signalling cascade during DNA replication.

**Fig. 4:**
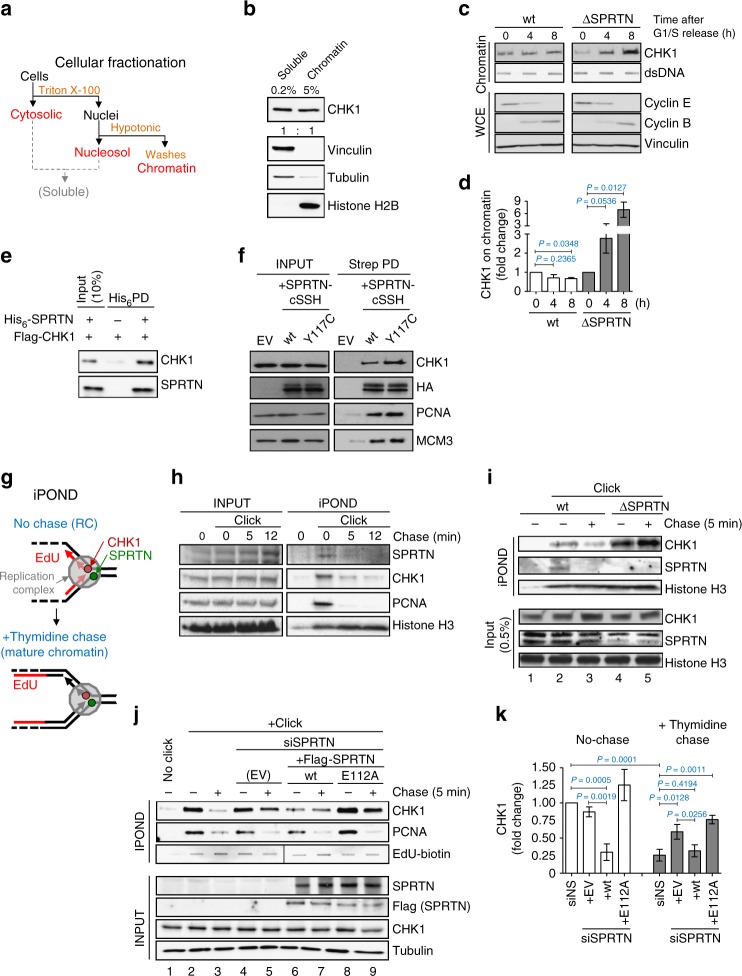
SPRTN protease evicts CHK1 from replicative chromatin. **a** Diagram of cellular fractionation into cytosolic, nuclear and chromatin fractions. The chromatin fraction was thoroughly washed with 1% NP-40 and 250 mM NaCl to isolate clean chromatin. **b** The cleanliness of the chromatin fraction was confirmed by fractionation protein markers. **c**, **d** SPRTN deficiency leads to accumulation of tightly bound CHK1 on chromatin during the S-phase progression. HeLa-wt and HeLa-ΔSPRTN cells were arrested at the G1/S boundary by a double thymidine block and then released to progress through S-phase. **c** Proteins tightly bound to DNA were isolated by a modified RADAR assay and the total content of CHK1 tested by immunoblotting. Double-stranded DNA (dsDNA) was used as loading control. Cyclin E and cyclin B from whole cell extracts (WCE) were used as cell cycle markers. **d** Quantification of CHK1 in (**c**). Mean ± SEM; *n* = 2, two-tailed Student's *t*-test. **e** SPRTN and CHK1 interact physically in vitro. Purified CHK1 co-precipitated with recombinant His_6_-tagged SPRTN on Ni-NTA beads. Immunoblots represent three replicates. **f** SPRTN and CHK1 interact physically in vivo. CHK1 co-precipitated with SPRTN from lysates of Flp-In HEK293 T-REx cells expressing either SPRTN-wt or SPRTN-Y117C fused to a cSSH (Strep-strep-HA) tag. Immunoblots represent three replicates. EV: empty vector, PD: pull down. **g**, **h** iPOND analysis revealed the presence of SPRTN and CHK1 proteins at nascent DNA (0 min) but not on mature chromatin (5–12 min after a thymidine chase). Cells were treated with EdU for 10 min to label nascent DNA, and then chased or not with thymidine. PCNA and histone H3 were used as replication fork and loading controls, respectively, *n* = 3. **i** HeLa-ΔSPRTN cells exhibit increased retention of CHK1 on mature DNA compared with SPRTN-proficient (wt) cells. **j**, **k** SPRTN protease is necessary for the efficient eviction of CHK1 from replicative chromatin. Expression of SPRTN-wt, but not of the protease inactive variant SPRTN-E112A, in SPRTN knock-down cells reduced the amount of CHK1 on replicative chromatin and reversed the accumulation of CHK1 in chased chromatin. Mean ± SEM; *n* = 3, two-tailed Student's *t*-test. Source data for (**b**–**f**, **h**–**k**) are provided as a Source Data file

To this end, we performed a series of in vitro and in vivo experiments to show that CHK1 is a direct substrate of the SPRTN protease. First, by cell fractionation we demonstrated that a small but significant fraction of CHK1 is constantly bound to chromatin (Fig. 4a, b). Additionally, CHK1 significantly hyper-accumulated on replicative/S-phase chromatin isolated under stringent and denaturing conditions in SPRTN-deficient cells during S-phase progression (Fig. 4c, d), also indicating that a portion of CHK1 is strongly attached to chromatin. Secondly, in vitro pull-down experiments using purified SPRTN and CHK1 proteins confirmed that the interaction between these two proteins is direct (Fig. 4e). Thirdly, further co-immunoprecipitation experiments from HEK293 cells expressing SPRTN-wt or the RJALS patient protease defective variant SPRTN Y117C, confirmed that both form a physical complex with CHK1, PCNA and MCM3^56^, further indicating that SPRTN and CHK1 are components of the DNA replication machinery (Fig. 4f). Finally, using iPOND to directly monitor SPRTN-dependent CHK1 dynamics on replicative chromatin (Fig. 4g, h), we demonstrated that both CHK1 and SPRTN are present at/around sites of DNA replication forks (nascent DNA) and travel with the fork, as confirmed by their absence from mature chromatin (after the thymidine chase) (Fig. 4h). Importantly, inactivation of SPRTN led to a strong accumulation of CHK1 on both nascent and mature chromatin (Fig. 4i, compare lanes 2 and 3 with 4 and 5).

In light of these observations, we took advantage of iPOND to assess if SPRTN proteolysis plays a role in CHK1 release/eviction from sites of replicative chromatin. We analysed the abundance of CHK1 by iPOND in SPRTN-knocked-down cells expressing an empty vector (EV), SPRTN-wt or the SPRTN protease-deficient variant E112A (Fig. 4j, k). Although both SPRTN variants were equally expressed, SPRTN-wt strongly evicted CHK1 from nascent (DNA replication sites) and mature chromatin, but SPRTN-E112A did not (compare lanes 4, 5 to 6, 7 or 8, 9). A similar effect was observed on total chromatin isolated by biochemical fractionation (Supplementary Fig. 4b, c). Altogether, these results support the model that the SPRTN-CHK1 complex is involved with the DNA replication machinery and that SPRTN proteolysis evicts CHK1 from replicating chromatin during DNA synthesis.

### SPRTN protease cleaves the C-terminal part of CHK1

**Fig. 5:**
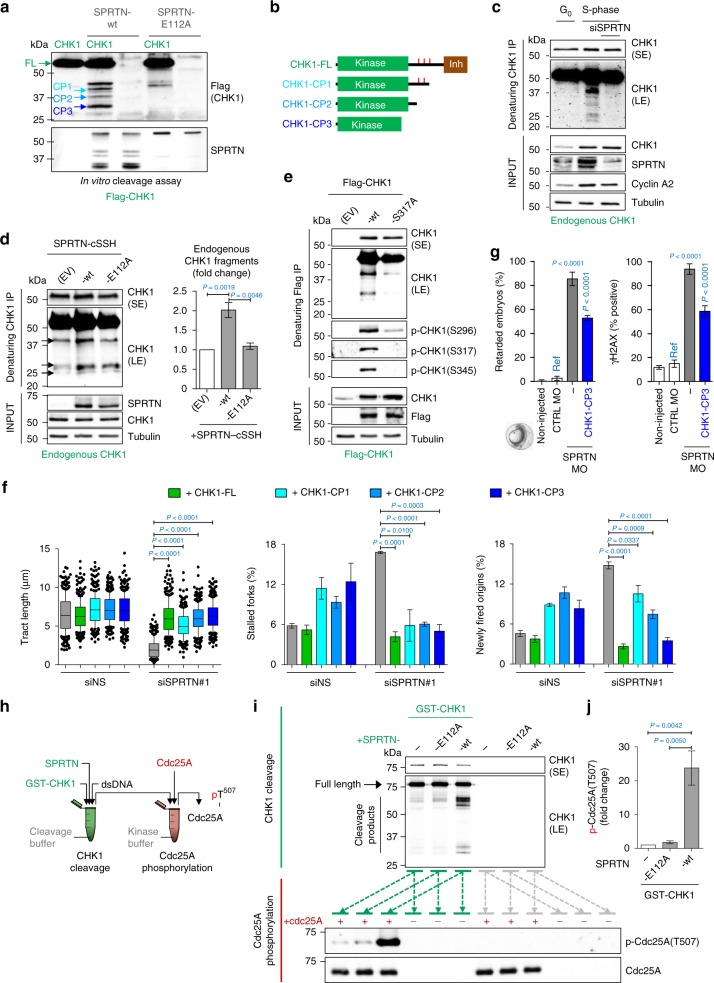
SPRTN cleaves CHK1 and releases kinase-active CHK1 fragments. **a** Purified Flag-CHK1 was incubated with recombinant SPRTN-wt or SPRTN-E112A (a protease-dead variant). Anti-Flag immunoblotting detected N-terminal CHK1 fragments released by the SPRTN proteolytic activity, referred to here as CHK1 Cleavage Products (CPs) -1, -2 and -3 and indicated by blue arrows. Representative immunoblots from three replicates. **b** Graphical representation of the three CPs released by SPRTN activity. The approximate size of these CPs was estimated from the immunoblots; CHK1 amino acids 1–338, 1–293 and 1–237, respectively. Red ticks represent the locations of Ser296, Ser317 and Ser345. Inh: autoinhibitory domain. **c**, **d** Endogenous CHK1 from T24 cells synchronised in G_0_- or S-phase (**c**) or from HEK293 cells ectopically expressing SPRTN-wt or SPRTN-E112A (**d**) was immuno-purified under denaturing conditions using a CHK1 antibody against an N-terminal CHK1 epitope. SE: short exposure, LE: long exposure. Quantification of the CHK1 fragments for (**d**). Mean ± SEM; *n* = 3, two-tailed Student's *t*-test. **e** Lysates from HEK293 cells ectopically expressing Flag-CHK1-wt or Flag-CHK1-S317A were denatured and CHK1 was then immuno-purified. Representative immunoblots from three replicates. **f** Ectopic expression of CHK1-CPs or CHK1-FL (full-length) in SPRTN-inactivated HEK293 cells. Left: mean ± 25–75 percentile range (box) ± 10–90 percentile range (whiskers); centre and right: mean ± SEM. >100 DNA fibers were analysed per condition and experiment; *n* = 3 experiments, two-tailed Student's *t*-test. **g** Ectopic expression of CHK1-CP3 in zebrafish embryos. Ref: reference value for statistics. Mean ± SEM; *n* = 3, two-tailed Student's *t*-test. **h** Graphical description of experimental setting. In the first reaction, GST-CHK1 was mixed with SPRTN-E112A or SPRTN-wt to generate CHK1 fragments (o/n, 37 °C); in the subsequent reaction, the product of the first reaction was mixed with Cdc25A in a kinase buffer to induce its phosphorylation (30 min, 37 °C). **i** Top panel: cleavage of CHK1 by SPRTN-wt after the first reaction. Bottom panel: phosphorylation of Cdc25A by the products of the first reaction. **j** quantification of phospho-Cdc25A in (**i**). Experiment was repeated 3 times with similar results. Source data for (**a**, **c**–**g**, **i**, **j**) are provided as a Source Data file

As SPRTN directly interacts with (Fig. 4e) and evicts CHK1 from sites of DNA replication in a protease-dependent manner (Fig. 4g–k), we investigated whether SPRTN proteolytically cleaves CHK1. Purified Flag-CHK1 from HEK293 cells was incubated with SPRTN-wt or a protease-deficient variant (E112A) in vitro in the presence of dsDNA, an activator of SPRTN metalloprotease activity (Fig. 5a). SPRTN-wt but not SPRTN-E112A cleaved the C-terminal part of CHK1, generating at least three N-terminal CHK1 fragments [Cleaved Products (CPs) 1, 2 and 3], as detected by anti-Flag antibody (Fig. 5a, b).

Similar N-terminal CHK1 fragments were observed on endogenous CHK1 in S-phase but not in G_0_-phase of the cell cycle in T24 urinary bladder carcinoma cell line or in HEK293 cells over-expressing SPRTN-wt (Fig. 5c, d). Importantly, endogenous CHK1 cleaved fragments were completely SPRTN protease dependent. Consistent results were observed with ectopically expressed CHK1 in vivo (Supplementary Fig. 5a). However, the formation of these cleavage products was reduced in cells overexpressing the CHK1 variant S317A (Fig. 5e) or treated with an ATR inhibitor (Supplementary Fig. 5b). These results further support the premise that CHK1 is a substrate of SPRTN during S-phase of the cell cycle, and that upstream ATR-phosphorylation assists CHK1 cleavage in vivo.

SPRTN is a DNA-dependent but amino-acid sequence unspecific protease. Its known substrates histones are cleaved in disordered protein regions in the vicinity of arginine, lysine and serine residues^25^. The C-terminal part of CHK1 is also loosely structured and serves as an inhibitory domain of the CHK1 kinase active centre located at Asp130 in the N-terminal part^52,57^. To better understand how SPRTN cleaves CHK1, we created the CHK1-7KA/3RA/8SA variant, where 7 lysines, 3 arginines and 8 serines were replaced with alanine in the predicted CP2 and CP3 cleavage sites. These mutations increased local disorder probability in mutated CHK1-7KA/3RA/8SA variant in the vicinity of SPRTN cleavage sites (CP2 and 3) (Supplementary Fig. 5c, d). We observed strikingly stronger SPRTN-mediated cleavage of CHK1-7KA/3RA/8SA variant in comparison to CHK1-wt (Supplementary Fig. 5e), likely because these mutations increased loosely structured protein regions and decreased the overall protein stability of CHK1, making it a better SPRTN substrate^25^.

### SPRTN protease is a bona-fide CHK1 activator

To investigate if the SPRTN-generated N-terminal CHK1 fragments are kinase active, we first estimated the size of SPRTN-dependent CHK1 cleaved fragments by their molecular weight and designed constructs mimicking these CPs for expression in vertebrates (Fig. 5b and Supplementary Fig. 5f). Interestingly, these CHK1 fragments exhibited ~5–10-fold more potent kinase activity than CHK1 full-length—even when the latter had been primed/activated by ATR in the cells—and they were able to phosphorylate SPRTN or the well-characterised CHK1 substrate Cdc25A in vitro (Supplementary Figs. 5g–i and 6a). As expected, while the kinase activity of full length CHK1 isolated from HEK293 cell treated with ATR inhibitor was affected, the kinase activity of the N-terminal CHK1 fragments was refractory to ATR inhibitor (Supplementary Fig. 6a).

Most notably, the N-terminal CHK1 products were fully functional in vivo, as their ectopic expression was able to restore DNA replication fork velocity and suppress new origin firing and fork stalling in SPRTN-depleted cells (Fig. 5f). Importantly, the recovery of DNA replication velocity with the N-terminal CHK1 product CP3 was sensitive to the CHK1 kinase inhibitor UCN-01 but resistant to the ATR kinase inhibitor VE-821 (Supplementary Fig. 6b–d), and this rescue was completely dependent on the kinase active centre Asp130 (Supplementary Fig. 6d). These results further suggest that the N-terminal CHK1 fragments, which mimic the cleaved CHK1 fragments generated by SPRTN proteolysis, are kinase active and independent of ATR.

Similarly, expression of CHK1-CP3 corrected developmental defects and suppressed DNA damage in SPRTN-depleted zebrafish embryos to the same extent as full-length CHK1-wt (Fig. 5g, compare with Fig. 2f, g).

To check if SPRTN proteolysis is a bona-fide CHK1 activator in an ATR-independent setting, we designed an in vitro assay with purified proteins from *Escherichia coli* expression system: GST-CHK1, SPRTN-wt, SPRTN-E112 and Cdc25A (Fig. 5h). The phosphorylation status of threonine 507 on Cdc25A was used as a read-out for CHK1 activation.

Consistent with previous reports, the unmodified full-length GST-CHK1 only phosphorylated Cdc25A with an extremely low efficiency^57^. The complete inability of recombinant SPRTN to phosphorylate Cdc25A by itself ruled out the possibility that unspecific kinases from *E. coli* had been co-purified with SPRTN (Fig. 5i, j and Supplementary Fig. 6e).

Remarkably, when CHK1 was cleaved by SPRTN in vitro (Fig. 5h, i; top panel) its kinase activity increased by more than 20-fold (Fig. 5i—bottom panel, j). Catalytic proteolysis by SPRTN was essential for this direct activation of CHK1 since the protease inactive SPRTN-E112A mutant was unable to cleave CHK1 (Fig. 5i, upper gel) and stimulate its phosphorylating activity (Fig. 5i, lower gel and Supplementary Fig. 6e).

Altogether, these results suggest (i) that the activation of CHK1 kinase activity is due to the SPRTN-dependent proteolytic cleavage of its C-terminal part and (ii) that even a limited proteolysis of CHK1 by SPRTN, generating a small fraction of cleavage products as that observed in human cell lines or in a test tube, is sufficient to trigger a potent CHK1 activation.

### CHK1 stimulates recruitment of SPRTN to chromatin

**Fig. 6:**
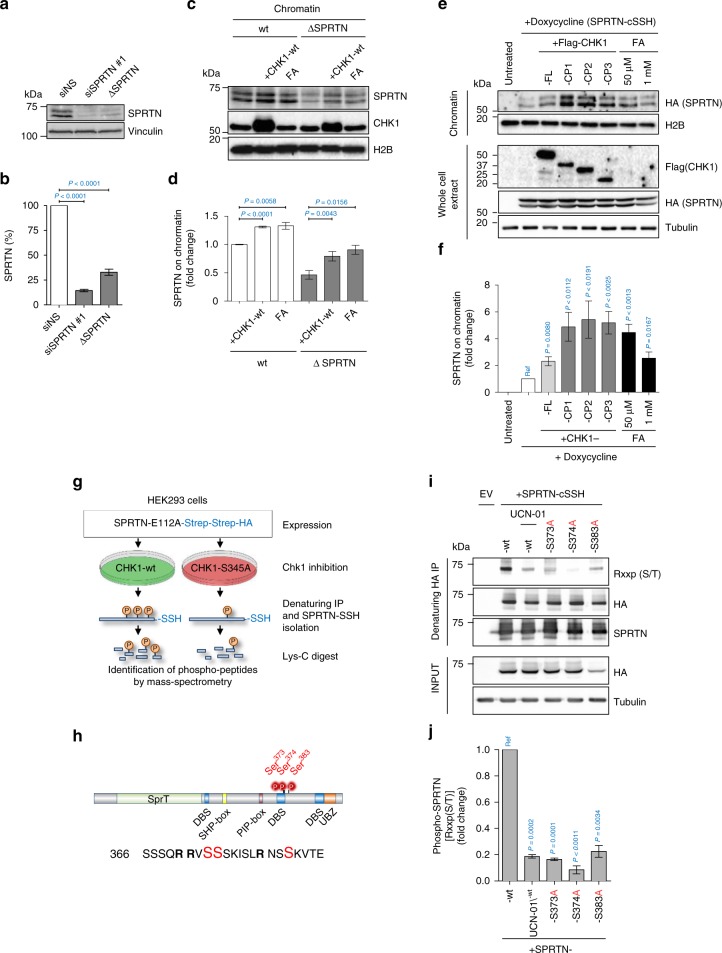
CHK1 phosphorylates SPRTN and regulates its recruitment to chromatin. **a**, **b** siRNA SPRTN-depleted HEK293 or haploinsufficient SPRTN HeLa cells (ΔSPRTN) still contain a residual amount of SPRTN (10, 30%, respectively). Data are representative immunoblots (**a**) and quantification of SPRTN normalised to vinculin (**b**). Mean ± SEM; *n* = 3, two-tailed Student's *t*-test. **c**, **d** CHK1 promotes SPRTN recruitment on chromatin. HeLa-wt or -ΔSPRTN cells ectopically expressing CHK1-wt, or treated with formaldehyde (FA) as a positive control, were fractionated and endogenous SPRTN protein level was assessed in the chromatin fraction. Residual SPRTN in ΔSPRTN cells were also recruited to chromatin. Representative immunoblots (**c**) and quantification of SPRTN on chromatin (**d**). Mean ± SEM; *n* = 3, two-tailed Student's *t*-test. **e**, **f** Overexpression of CHK1-full length (FL) or N-terminal products (CP1, 2 or 3) promote SPRTN retention on chromatin. Doxycycline-inducible stable cells expressing SPRTN-wt-cSSH cells were fractionated and SPRTN was assessed in chromatin fractions. Data are representative immunoblots (**e**) and quantification of HA (SPRTN) on chromatin (**f**). Mean ± SEM; *n* = 3, two-tailed Student's *t*-test. **g** Schematics to identify the CHK1 phosphorylation sites on SPRTN by Mass-Spectrometry. **h** Diagram depicting human SPRTN with the CHK1 phosphorylation sites on Ser 373, 374 and 383 identified in this study. SPRTN domain and motifs; DBS: DNA binding site; SHP box: p97 interacting region; PIP box: PCNA interacting peptide; UBZ: ubiquitin binding domain. Sequence below (from SwissProt: Q9H040) shows the location of these sites highlighted in red and amino acids surrounding the region. **i**, **j** The degree of phosphorylation at a CHK1 consensus target sequence is diminished in the phospho-deficient SPRTN variants. Different SPRTN variants were expressed in HEK293 cells and purified under denaturing conditions. An antibody recognizing the epitope Rxxp (S/T) (phospho-Ser/Thr preceded by Arg at position -3) was used to visualise CHK1 phosphorylated targets. The CHK1 inhibitor UCN-01 was used as a control. Data are representative immunoblots (**i**) and quantification of CHK1-mediated phosphorylation of SPRTN normalised to total SPRTN protein level in denatured sample (**j**). Mean ± SEM; *n* = 3, two-tailed Student's *t*-test. Source data for (**a**–**f**, **i**, **j**) are provided as a Source Data file

Our results so far indicate that SPRTN is needed for proper activation of CHK1 during physiological DNA replication (Fig. 1). This CHK1 activation occurs by both (i) SPRTN proteolysis-dependent eviction of CHK1 from replicative chromatin (Fig. 4g–k) and (ii) by simultaneous cleavage of the inhibitory/regulatory C-terminal parts of CHK1 (Fig. 5a–e, h–j). Paradoxically, ectopic expression of CHK1 restored various tested phenotypes of SPRTN-deficiency both in human cells and zebrafish embryos (Figs. 2 and 5f, g and Supplementary Fig. 6b–d). As siRNA and MO-mediated SPRTN depletion is never 100% efficient, we speculated that ectopic CHK1 expression stimulates, by phosphorylation (Supplementary Fig. 5g), the remaining SPRTN to process DPCs during DNA synthesis. Indeed, biochemical analysis demonstrated that SPRTN-inactivated cells still retain approximately 20–30% of SPRTN protein (Fig. 6a, b). To investigate this possible cross-talk between SPRTN and CHK1, we monitored SPRTN recruitment to chromatin after ectopic CHK1 expression (Fig. 6c, d). The amount of SPRTN on chromatin was substantially increased after CHK1 overexpression, suggesting that CHK1 induces SPRTN recruitment to chromatin. Importantly, the residual endogenous SPRTN in Δ-SPRTN cells (≈30% of control cells; Fig. 6a, b) hyper-accumulated on chromatin after CHK1 overexpression, almost to the same level as in untreated wild-type cells (Fig. 6c, d). To further support these results, we induced SPRTN-SSH expression in doxycycline-inducible HEK293 cells and ectopically co-expressed CHK1-wt or the N-terminal CHK1 cleaved fragments (CP1, 2 and 3). Ectopic expression of CHK1-wt stimulated SPRTN-SSH accumulation on chromatin. Strikingly, ectopic expression of the N-terminal CHK1 fragments additionally enhanced the SPRTN-SSH recruitment to chromatin (Fig. 6e, f), and also phosphorylated SPRTN much more than full length CHK1 (Supplementary Fig. 5g, h). These results may explain why the ectopic expression of CHK1-wt or its N-terminal fragments restores the phenotypes arising from SPRTN-deficiency in human cell lines and zebrafish embryos.

### CHK1 phosphorylates the C-terminal regulatory part of SPRTN

To identify CHK1 phosphorylation sites on SPRTN in vivo, we performed mass spectrometry. SPRTN-E112A-SSH was ectopically expressed in HEK293 cells co-transfected with either CHK1-wt or the kinase-defective CHK1-S345A, and anti-SSH precipitates were isolated under denaturing conditions (Fig. 6g and Supplementary Fig. 7a). The SPRTN-E112A protease dead mutant was used to avoid auto-cleavage activity^25^ (Fig. 5a, lower panel). Mass spectrometry analysis identified three main CHK1 phosphorylation sites on SPRTN—serines 373, 374 and 383 (Fig. 6h, Supplementary Fig. 7b, c and Supplementary Data 1)—located in the C terminal part of SPRTN. These sites correspond to a consensus target motif of CHK1 (R/K-x-x-p(S/T))^58^. To validate our results, we isolated SPRTN-wt-SSH or -S373A, -S374A and -S383A variants from HEK293 cells treated with DMSO or the CHK1 inhibitor UCN-01. SPRTN isolates were analysed by Western blot using an antibody that recognises CHK1 consensus sites (Fig. 6i, j). SPRTN-wt was phosphorylated in control cells but this phosphorylation signal was strongly decreased when cells were treated with UCN-01. Importantly, SPRTN phosphorylation was strongly reduced (S373A or S383A) or almost completely abolished (S374A) even when only one of the three identified serines (S373, S374 and S383) was mutated. This suggests that the C-terminal, regulatory part of SPRTN is phosphorylated by CHK1 at these specific serines under steady state conditions.

### SPRTN-CHK1 cross-activation loop

**Fig. 7:**
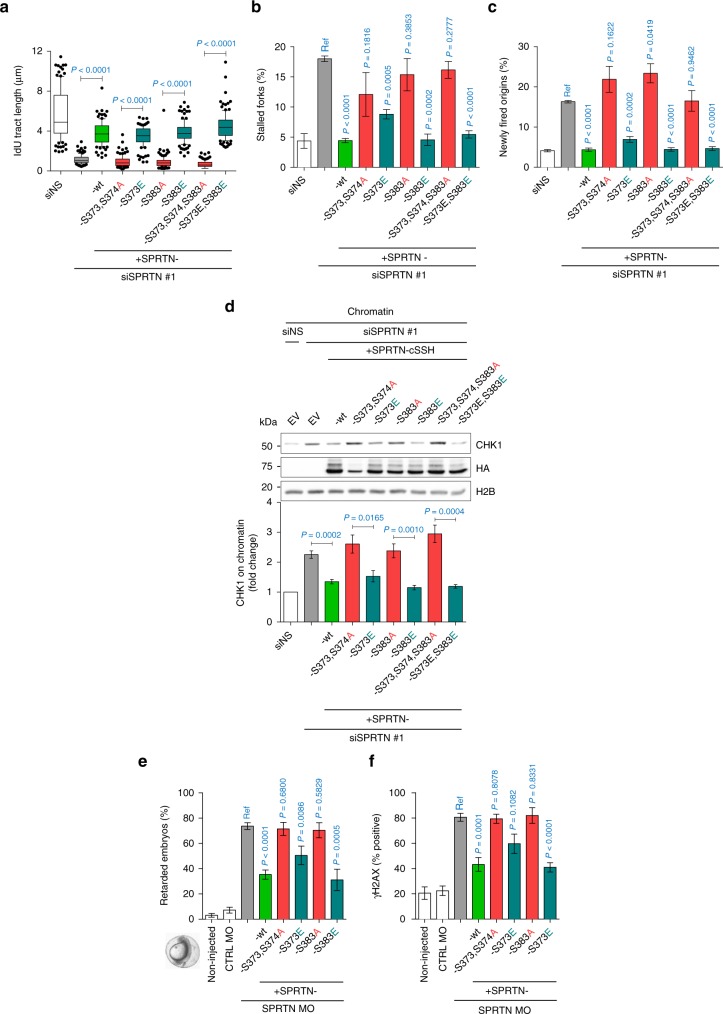
CHK1 targeted phosphorylation of SPRTN is essential for its biological function. **a**–**c** DNA replication analysis based on DNA fibers showing that SPRTN phospho-defective mutants (S to A) are functionally impaired and unable to rescue DNA replication fork velocity (**a**), suppress new origin firing (**b**) and decrease fork stalling (**c**) in SPRTN-depleted HEK293 cells, whereas SPRTN phospho-mimetic mutants (S to E) are functional and restore normal DNA replication phenotype. Left: mean ± 25–75 percentile range (box) ± 10–90 percentile range (whiskers); right: mean ± SEM. >100 DNA fibers were analysed per condition and experiment; *n* = 3 experiments, two-tailed Student's *t*-test. **d** SPRTN phospho-defective mutants failed to promote the eviction of CHK1 from chromatin. Chromatin fractions from HEK293 cells were immunoblotted to show the effect of SPRTN variants on CHK1 release from chromatin. Data are representative immunoblots (top) and quantification of CHK1 on chromatin fraction normalised to histone H2B (bottom). Mean ± SEM; *n* = 3 experimental replicates, two-tailed Student's *t*-test. **e**, **f** SPRTN phospho-defective mutants were unable to rescue the zebrafish embryo developmental retardation (**e**) and DNA damage (**f**, γH2AX positive signal) induced by SPRTN deficiency. SPRTN-depleted embryo cells (SPRTN MO) were co-injected with plasmids for the expression of SPRTN variants. >70 embryos were scored per experiment. Mean ± SEM; *n* = 3 independent experiments, two-tailed Student's *t*-test. Source data for (**a**–**f**) are provided as a Source Data file

To biologically validate the biochemical evidence that CHK1 phosphorylates SPRTN and thus regulates SPRTN’s function, we depleted SPRTN in HEK293 cells and tested the effects of ectopically-expressing variants of SPRTN that either could or could not be phosphorylated by CHK1. SPRTN-wt and SPRTN-phospho-mimetic variants S373E and S383E restored DNA replication defects (Fig. 7a–c) and promoted CHK1 release from chromatin (Fig. 7d). Conversely, SPRTN phospho-defective variants S373A/374A and SPRTN-S383A completely failed to restore these phenotypes in SPRTN-depleted cells. Similar findings were observed in zebrafish embryos (Fig. 7e, f). These data suggest that CHK1-mediated phosphorylation of SPRTN is important for SPRTN’s function in vivo.

**Fig. 8:**
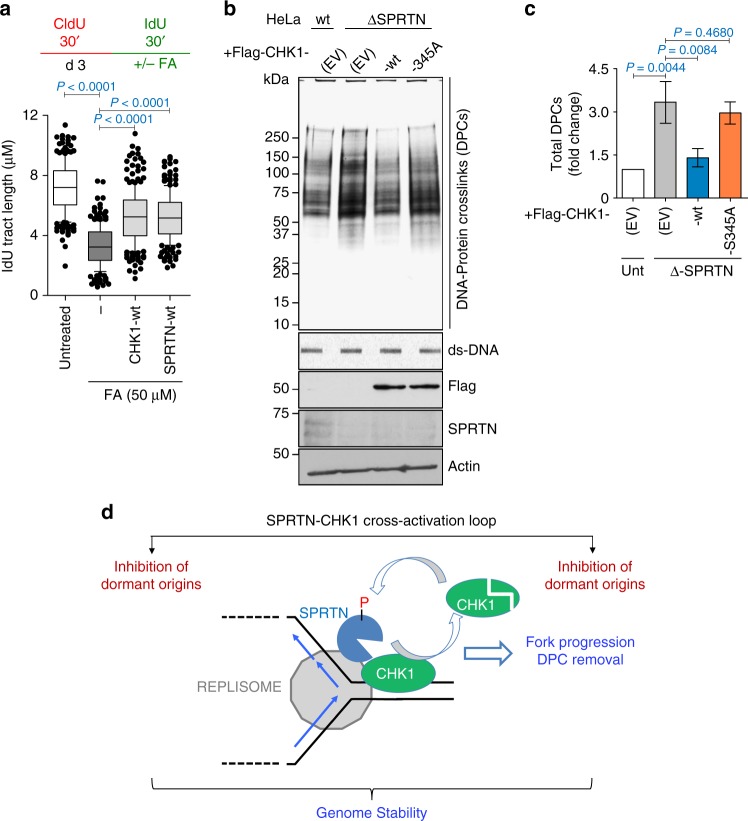
CHK1 and SPRTN promote replication fork progression by removing DPCs. **a** Ectopic expression of CHK1-wt or of SPRTN-wt promotes replication fork progression over FA-induced DPCs in HEK293 cells. Upper panel shows the strategy for DNA fiber assay. Lower graph plots the quantification of IdU tract length. >100 DNA fibers were analysed per condition and experiment. Mean ± 25–75 percentile range (box) ± 10–90 percentile range (whiskers), *n* = 3 experiments, two-tailed Student's *t*-test. **b**, **c** Overexpression of CHK1-wt, but not of CHK1-S345A, reduces the amount of DPCs in HeLa-ΔSPRTN cells. DNA protein crosslink extracts were visualised by silver staining (**b**). ds-DNA blot is shown as a loading control. The immunoblots for Flag, SPRTN and actin were prepared from whole cell lysates. Data are representative immunoblots (**b**) and quantification of CHK1 on chromatin fraction normalised to histone H2B (**c**). Mean ± SEM; *n* = 3 experimental replicates, two-tailed Student's *t*-test. **d** Model for the SPRTN-CHK1 cross-activation loop. During steady-state DNA replication, SPRTN evicts CHK1 from replicative chromatin and stimulates CHK1 activity. In turn, CHK1 phosphorylates SPRTN on C-terminal serines. CHK1 activity enhances the recruitment of SPRTN to chromatin and its activity towards DPC proteolysis. This steady-state SPRTN-CHK1 cross-activation loop enables normal physiological replication fork velocity, inhibits unscheduled replication origins firing, stabilises replication forks and removes DPCs to safeguard genome stability. Source data for (**a**–**c**) are provided as a Source Data file

To test our model in which a SPRTN protease-CHK1 kinase cross-activation loop processes DPCs in front of the DNA replication fork, we measured the progression of DNA replication fork over FA-induced DPCs by DNA fiber assay (Fig. 8a). Transient and low dose FA treatment strongly reduced DNA replication fork velocity, but this could be significantly reversed by the expression of either CHK1-wt or SPRTN-wt. This further supports that both SPRTN and CHK1 prevent DPC-induced DNA replication fork slowing and stalling.

Similarly, ectopic expression of CHK1-wt, but not of the kinase deficient variant CHK1-S345A, reduced the levels of DPCs in Δ-SPRTN HeLa cells to roughly the same level as in wild-type cells (Fig. 8b, c). Altogether, these results support our model (Fig. 8d) that a SPRTN-CHK1 cross-activation loop works during physiological DNA replication to prevent DNA replication stress and preserve genomic stability.

## Discussion

A large body of evidence suggests that CHK1 is essential during unperturbed DNA synthesis when the amount of ssDNA required to activate the canonical ssDNA-ATR-CHK1 signalling cascade is limited^15,16,19,20,59^. In recent years, a growing number of additional factors that activate ATR and CHK1 during replication-associated DNA damage signalling have been described, such as TopBP1, ETAA1 and Claspin^21,22^, as have DNA damage-independent sources of ATR activation, like nuclear envelope mechanical stress^60^. Despite this, the crucial question remains unanswered: how CHK1 is released from chromatin and activated during unperturbed DNA replication.

Our data and published literature^12,13^ suggest that ATR-mediated phosphorylation is needed for efficient CHK1 cleavage, chromatin release and activation of the ATR-CHK1 signalling cascade and that SPRTN-dependent proteolysis is important to sustain functional CHK1 activity during DNA replication. SPRTN protease plays an important role in the activation of CHK1 kinase during unperturbed DNA synthesis by removing the C-terminal inhibitory domain of CHK1.

By using an in vitro reconstitution system of CHK1 activation by SPRTN proteolysis, we additionally supported the proposed model. This result also demonstrates that even a modest CHK1 cleavage by SPRTN—observed on both endogenous or over-expressed CHK1 in vivo—is sufficient to strongly activate CHK1 kinase activity. As SPRTN cleaves CHK1 in a sequence-unspecific manner in its loosely-structured C-terminal region, the exact size of the N-terminal CHK1 fragments released is unimportant. This is demonstrated by the observation that all three predicted N-terminal CHK1 fragments are kinase active and able to restore all tested phenotypes in SPRTN-deficient human cells. Even SPRTN-deficiency phenotypes in Zebrafish embryos were rescued by CHK1-CP3 fragment. Our results are further supported by previous work which demonstrates the inhibitory role of the C-terminal part of CHK1 on CHK1 kinase activity^57,61^.

To ultimately prove the proposed model (Fig. 8d), a SPRTN variant unable to interact with CHK1 in vivo would be needed. Cells bearing SPRTN variants defective for CHK1 interaction are expected to suffer from DNA replication stress as the steady-state CHK1 activity would not be activated due to CHK1 chromatin retention.

Our results also partially address how SPRTN is regulated under physiological conditions by means of phosphorylation. The regulation of SPRTN has a considerable interest given its pleiotropic protease activity. SPRTN is physically present at DNA replication forks and the SPRTN gene is essential in metazoan from early embryogenesis. It has been previously proposed that ssDNA promotes SPRTN-dependent substrate cleavage while dsDNA induces SPRTN auto-cleavage to negatively regulate SPRTN activity^26^. However there are two arguments against this model: (i) SPRTN is an essential enzyme for DPC repair during DNA replication, yet DPCs prevent the helicase–polymerase uncoupling and the ssDNA formation that would be required to activate SPRTN, and (ii) SPRTN protease activity towards DNA-binding substrates is also stimulated by dsDNA^25,27^, as additionally demonstrated in this manuscript using CHK1 as an in vitro substrate.

Our results suggest a model in which SPRTN protease activity is regulated by CHK1 kinase activity during physiological DNA replication. CHK1 constitutively phosphorylates SPRTN at the C-terminally located serines S373, S374 and S383, which is essential for DNA replication and embryonic development. Further investigation should elucidate the phosphorylation dependant regulation of SPRTN.

In summary, the DNA-replication coupled SPRTN-CHK1 cross-activation loop proposed here opens a new avenue in our attempt to better understand genome instability associated with DNA replication and places the protease SPRTN as a new potential therapeutic target in cancer therapy based on targeting DDR checkpoints^62^.

## Methods

Key resources used in this study are listed and described in Supplementary Table 1. Oligonucleotide sequences are listed in Supplementary Table 2.

### Experimental models and cell culture

U2OS, HeLa and HEK293 human cells were maintained in DMEM (Sigma-Aldrich) supplemented with 10% FBS (Gibco) and 100 I.U./mL penicillin/0.1 mg/mL streptomycin at 37 °C in a humidified incubator with 5% CO_2_, and tested for mycoplasma contamination. CRISPR partial knockout Δ-SPRTN HeLa cells^25^ were maintained as above. Media for doxycycline-inducible stable HEK293 Flp-In TRex SPRTN-wt or SPRTN-Y117A cell lines^25^ was additionally supplemented with 15 μg/mL blasticidin/100 μg/mL hygromycin. Wild-type zebrafish (*Danio rerio* EK and AB strains) were kept under standardised conditions in a circulating water system on a 14 h light and 10 h dark cycle. All procedures involving zebrafish embryos adhered to current European regulations for the use of animals and were approved by the local authorities of Ulm University.

### Bacterial strains

Competent *E. coli* DH5alpha (Invitrogen) and *E. coli* Rosetta2 (Merck; Novagen), used for cloning/plasmid amplification or for protein expression, respectively, were transformed with plasmid and grown in LB medium at 37 °C.

### Subcloning and site-directed mutagenesis

The pCINeo/CMV-Flag-CHK1-wt plasmid^12^ was used for expression in mammalian cells and to generate all CHK1 variants by site-directed mutagenesis. For the expression of CHK1 in Zebrafish, CHK1 variants were subcloned into pCS2-GFP vector by PCR from previous plasmids and EcoRI/XbaI insertion. Site-directed mutagenesis was performed by plasmid PCR using specific mutagenic primers (sequences in Supplementary Table 2) designed by the quick-change primer design software (Invitrogen) and AccuPrime Pfx DNA-polymerase (Invitrogen). Mutations were then verified by sequencing at Source BioScience, Oxford, UK. CHK1-7KA/3RA/8SA variant in pCINeo/CMV-Flag plasmid was created by gene synthesis (Biomatik).

### RNA interference and plasmid transfection

siRNA (sequences in Supplementary Information, Supplementary Table 2) transfections were performed using Lipofectamine RNAiMax reagent (Invitrogen) according to manufacturer’s instructions. Depletion was assayed 72 h post-transfection. Plasmid (Supplementary Information, Supplementary Table 1) transfections were performed using either FuGene HD reagent (Promega) following the manufacturer’s instructions or by PEI method. Protein expression was assayed 12–48 h post-transfection.

### Denatured whole cell extracts

Cells were collected, washed three times in ice-cold PBS and lysed in RIPA buffer (50 mM Tris–HCl, pH 7.4, 150 mM NaCl, 1% NP-40, 0.5% SDS, 0.1% SDS, 3 mM EDTA, 20 mM N-ethylmaleimide (NEM), 1 mM DTT, protease inhibitors [1 μg/mL Chymostatin, 1 μg/mL Aprotinin, 1 μg/mL Leupeptin, 1 mM PMSF] and phosphatase inhibitors [1 mM Na_3_VO_4_×2H_2_O, 1 mM Na_4_P_2_O_7_, 20 mM NaF]). Extracts were then sonicated (20 cycles; 30/30 s on/off) in a Bioruptor Plus sonicator (Diagenode).

### Cell fractionation

Cells were incubated in 2× volumes of Buffer A (10 mM HEPES, pH 7.4; 10 mM KCl; 1.5 mM MgCl_2_; 340 mM Sucrose; 10% Glycerol; 1 mM DTT; 5 mM EDTA; 20 mM NEM; protease and phosphatase inhibitors and 0.1% Triton-X100) on ice for 10 min. Sample was spun (350*g*, 4 min, 4 °C) and the supernatant was collected as the cytosolic fraction. Nuclei were then washed once with Buffer A without Triton-X100 and burst in 2× volumes of hypotonic Buffer B (3 mM EDTA; 0.2 mM EGTA; 1 mM DTT; 10 mM NEM; protease and phosphatase inhibitors) on ice for 5 min. NaCl to 250 mM and NP-40 to 0.6% were added and sample rotated for 10 min. After centrifugation at 2500*g* for 5 min, the supernatant was collected as the nuclear soluble fraction. The pellet was washed twice with Buffer B with 0.6% NP-40 and 250 mM (first wash) or 150 mM (second wash) NaCl, and this was considered as the chromatin fraction. For chromatin nuclease extracts, chromatin fraction was washed in Benzonase buffer (50 mM Tris pH 7.5; 20 mM NaCl; 5 mM KCl; 3 mM MgCl_2_; protease and phosphatase inhibitors) and incubated in same buffer at 4 °C with 100 u/mL Benzonase (Millipore).

### Denaturing immunoprecipitation

Denatured extracts were first obtained by lysing cells expressing tagged protein in denaturing buffer (50 mM Tris, pH 7.4; 137 mM NaCl; 5 mM KCl; 1% SDS; 1 mM DTT; 5 mM EDTA; 20 mM NEM; protease and phosphatase inhibitors), heating at 95 °C for 5 min, shearing chromatin by 10–15 passes through a 22-gauge needle and sonicating—20 cycles; 30/30 s on/off—in a Bioruptor Plus sonicator (Diagenode), before been clarified by centrifugation at 15,000*g* for 5 min. 10% of supernatant was retrieved for input. Denatured extract was diluted 10-fold in IP buffer (50 mM Tris, pH 7.4; 150 mM NaCl; 10 mM NEM; protease and phosphatase inhibitors) with 1% Triton X-100 to quench SDS, and precleared with blank sepharose (IBA) and 2 U avidin for 1 h. After incubation for 4 h to overnight with Strep-Tactin sepharose (IBA) or Anti-Flag M2 affinity beads (Sigma-Aldrich), extract was washed twice with IP buffer containing 500 mM NaCl and 1% Triton, and 3 times with IP buffer containing 137 mM NaCl and no detergents. Samples were finally eluted in 2× Laemmli buffer for 10 min at 95 °C.

### Protein co-immunoprecipitation

To isolate SPRTN-interacting proteins, non-denaturing whole cell lysates were prepared from corresponding HEK293 Flp-In TRex cell lines upon doxycycline induction of SPRTN-WT-cSSH (Strep-Strep-HA tag). In addition, cell lysates were also prepared from HEK293 cells transiently transfected with SPRTN-WT-cSSH and SPRTN-Y117-cSSH in order to compare the capturing efficiency between SPRTN-WT and SPRTN-Y117C (patient mutation), respectively. Cells were collected and lysed in non-denaturing lysis buffer [50 mM Tris pH 7.4; 150 mM NaCl; 1% Triton; 1 mM EDTA; 10 mM NEM; protease and phosphatase inhibitors] with 2 U avidin (IBA Cat# 2-0204-015) at 4 °C. Following centrifugation at 16,000*g* for 10 min, the supernatant was kept at 4 °C and the pellet was digested with Benzonase nuclease in Benzonase buffer (see above) at 37 °C for 1 h. Both supernatant and Benzonase digested fractions were pooled and 10% of this pooled fraction was stored at −20 °C for later use as input. The rest of the lysate was diluted 10 times in IP buffer [50 mM Tris, pH 8; 150 mM NaCl; 10 mM NEM; protease and phosphatase inhibitors] and precleared with blank sepharose (IBA GmbH) for 1 h at 4 °C on a rotating wheel. Precleared samples were then incubated with Strep-Tactin sepharose beads (IBA Cat# 2-1201-010) for 2 h at 4 °C, washed five times in IP buffer containing 0.05% NP-40 and eluted in 2× Laemmli buffer (62.5 mM Tris–HCl, pH 6.8; 2% SDS, 10% Glycerol, 2.5% β**-**mercaptoethanol; 0.001% Bromophenol blue) for 10 min at 95 °C.

### Western blotting

Samples for WB were prepared following standard methods. Briefly, protein concentration in cleared protein extracts was measured by Lowry (Bio-Rad) and samples were denatured in Laemmli buffer (95 °C, 5 min). SDS-PAGE was performed in TGS buffer. After transfer, membranes were blocked in TBST buffer with 5% milk (RT, 1 h), washed with TBST and incubated (overnight, 4 °C) with primary antibody (Supplementary Information, Supplementary Table 1) diluted 1:1000 in 2% BSA in TBST. Membranes were then washed four times in TBST, incubated (RT, 1 h) with HRP-conjugated secondary antibody (Supplementary Information, Supplementary Table 1) in TBST with 1% milk, and washed 4 times in TBST. Protein was then visualised using ECL substrates (Thermo Scientific and Bio-Rad) in a ChemiDoc MP system (Bio-Rad) or with X-ray film. Quantification of immunoblots was performed using ImageLab software (BioRad) or ImageJ. Specific protein level values were calculated relative to the corresponding loading control. Statistical significances of replicate measurements were analysed in GraphPad Prism.

### Isolation of proteins on nascent DNA: iPOND

To perform iPOND^25^, newly synthesised DNA (~2 × 10^8^ cells per condition) was labelled via incubation with 10 μM EdU for 10 min (in SPRTN-wt cells) or 15 min (in SPRTN depleted cells). For mature chromatin, cells were chased with thymidine for further 5 min. Chromatin fragmentation into 300–500 bp fragments was done in a Bioruptor Plus sonicator (Diagenode) (70 cycles, 30/30 s on/off). After conjugation of biotin to EdU by click chemistry^63^, proteins on EdU-labelled DNA were isolated with streptavidin-coupled agarose beads.

### DNA fiber assay

Asynchronous cells^25,31^ were labelled with 30 μM CldU for 30 min and then with 250 μM IdU for additional 30 min. For assay of genotoxic stress, IdU was incubated in the presence of drugs. DNA replication was inhibited with ice-cold PBS. Cells were lysed in 200 mM Tris–HCl pH 7.4, 50 mM EDTA and 0.5% SDS; DNA fibers were spread onto positively charged glass slides, fixed with 3:1 methanol: acetic acid (v/v), denatured with 2.5 N HCl, blocked with 2% BSA in PBS containing 0.001% Tween-20, and stained for 1 h at 37 °C with anti-BrdU that specifically recognize either CldU (clone BU1/75(ICR1)) or IdU (clone B44). After incubation, slides were washed three times with PBS containing 0.05% Tween-20 (PBS-T) and briefly blocked again. Anti-rat Cy3 (Jackson Immuno Research) and anti-mouse Alexa-Fluor 488 (Molecular Probes) were the respective secondary antibodies. Slides were washed 3 times with PBS-T, air-dried at RT and mounted with ProLong Gold antifade reagent (ThermoFisher). Microscopy was performed using a Leica DMRB microscope with a DFC360FX camera. Quantification of CldU- and IdU-labelled DNA tract lengths was done with ImageJ software on at least 100 fibers per condition in at least three independent experiments, converting arbitrary units to microns based on the microscope scale. Resulting “Tract length” values were represented in box graphs showing the mean (bar) with the 25–75 percentile range (box) and the 10–90 percentile range (whiskers) and statistically analysed using GraphPad Prism. Analysis of newly fired origins and of stalled forks were performed as above but on at least 400 fibers per condition tested, and data was represented as plot the mean ± SEM of at least three independent experiments.

### Metaphase spreads

HEK293 cells^31^ were treated with 100 ng/mL colcemid for 2 h, trypsinized, collected and incubated with 0.4% KCl at 37 °C for 15 min. Following the addition of fixative solution (3:1 methanol:acetic acid), cells were centrifugated at 1000*g* for 10 min, resuspended in 5 mL fixative solution, incubated at RT for 15 min, centrifuged, resuspended in 1 mL 100% acetic acid, incubated at RT for 10 min, centrifuged, and finally resuspended in 5 mL of fixative solution. Metaphase spreads were prepared by dropping the cell suspension from a height onto pre-wetted slides. The slides were air dried, stained with DAPI and examined by microscopy. Analysis were performed on at least 30 metaphases per sample.

### Immunofluorescence microscopy

Cells grown on round coverslips were washed once with ice-cold PBS, pre-extracted for 3 min with ice-cold pre-extraction buffer (25 mM HEPES pH 7.5; 50 mM NaCl; 1 mM EDTA; 3 mM MgCl_2_; 300 mM Sucrose; 0.5% Triton-X100) and fixed by adding methanol and 30 min incubation at −20 °C. Fixed cells were then washed three times with PBS, blocked with 3% BSA in PBS with 0.5% Tween-20 for 1 h at RT, incubated (90 min, RT) with the primary antibody, washed three times, and incubated with the corresponding secondary antibody (45 min, RT). Incubation with DAPI was done for 15 min at RT. Finally, coverslips were mounted onto clean glass slides with mounting media. Microscopy was performed using a Leica DMRB microscope with a DFC360FX camera.

### ssDNA foci

For the detection of single-stranded DNA (ssDNA) under non-denaturing condition^64^, cells were seeded on coverslips, transfected with siRNA and 24 h after, treated with 10 μM BrdU (Sigma-Aldrich) for further 48 h, and then pulse-labelled with 100 μM CldU for 30 min before being treated with replication interfering drugs. Cells were washed with PBS, pre-extracted, fixed and blocked as above. ssDNA was marked with anti-BrdU (clone B44; 1:100 dilution) for 1 h, then three washes with PBS, followed by secondary anti-mouse Alexa-Fluor 488 antibody (Molecular Probes, 1:1000 dilution) for 30 min and three washes. The primary and secondary antibody conjugates were then fixed with 4% formaldehyde in PBS for 15 min followed by another wash. DNA was then denatured in 1.5 N HCl for 30 min and washed with PBS. For the detection of replication foci after denaturation, sample was incubated with anti-CldU antibody (clone BU1, 1:100 dilution) for 1 h, washed twice with PBS, then with high salt buffer (PBS with 200 mM NaCl, 0.2% Tween-20 and 0.2% NP-40) for 15 min and then with PBS again, then incubated with anti-rat IgG Cy3 antibody (Jackson Immuno Research, 1:2000 dilution), and washed again. Finally, coverslips were mounted in mounting medium supplemented with 2× DAPI. Images were taken with a Leica DMRB microscope with a DFC360FX camera.

### DNA protein crosslink isolation and detection

DPCs were isolated using a modified Rapid Approach to DNA Adduct Recovery (RADAR) assay^65,25^. Briefly, 1–2 × 10^6^ cells were lysed in 1 mL of buffer containing 6 M guanidine thiocyanate (GTC); 10 mM Tris–HCl, pH 6.8; 20 mM EDTA; 4% Triton-X100; 1% Sarkosyl and 1% DTT. DNA was precipitated by adding 100% ethanol, washed three times in wash buffer (20 mM Tris–HCl, pH 6.8; 150 mM NaCl and 50% ethanol). DNA was then solubilized in 1 mL of 8 mM NaOH. The DNA concentration was quantified by treating a small aliquot of DNA with proteinase K (Invitrogen) for 3 h at 50 °C, followed by detection with PicoGreen dye (Invitrogen) according to manufacturer’s instructions. Equal dsDNA loading was confirmed by slot-blot immunodetection with an anti-dsDNA antibody (Abcam). Total DPCs after electrophoretic separation on polyacrylamide gels were visualised by silver staining using the ProteoSilver Plus Silver Stain Kit (Sigma-Aldrich) and specific proteins were detected by Western-blot.

### SPRTN purification

*Rosetta* (DE3) *E. coli* carrying the pNIC-ZB-SPRTN-wt or pNIC-ZB-SPRTN-E112A plasmids^25^ was resuspended in lysis buffer (100 mM HEPES, pH 7.5; 500 mM NaCl; 10% glycerol; 10 mM imidazole; 1 mM Tris(2-carboxyethyl) phosphine (TCEP); 0.1% dodecylmaltoside (DDM); 1 mM MgCl_2_ and protease inhibitor cocktail (Merck)) and sonicated. 1 U Benzonase nuclease was added to lysates before cell debris was pelleted by centrifugation. Lysates were applied to a Ni-sepharose IMAC gravity flow column, washed with two column volumes of wash buffer (50 mM HEPES, pH 7.5; 500 mM NaCl; 10% glycerol; 45 mM imidazole; 1 mM TCEP), and eluted in elution buffer (wash buffer but with 300 mM imidazole). Elution fractions were applied directly to a 5 mL Hitrap SP HP column (GE Healthcare), washed with wash buffer 2 (50 mM HEPES, pH 7.5; 500 mM NaCl; 1 mM TCEP) and eluted with elution buffer (wash buffer but with 1 M NaCl). The purification tag was cleaved by the addition of 1:20 mass ratio of His-tagged TEV protease during overnight dialysis into buffer A (20 mM HEPES, pH 7.5, 500 mM NaCl, 0.5 mM TCEP). Samples were concentrated by ultrafiltration using a 30 kDa molecular weight cut-off centrifugal concentrator and loaded onto size exclusion chromatography using a HiLoad 16/60 Superdex 200 column (GE Healthcare) at 1 mL/min in buffer A. Protein identities were verified by LC/ESI-TOF mass spectrometry^25^ and protein concentrations were determined by absorbance at 280 nm (Nanodrop) using the calculated molecular mass and extinction coefficients.

### In vitro cleavage of CHK1

Recombinant SPRTN wt or E112A mutant were purified in house from *E. coli*. Flag-CHK1 variants, wt or 7KA/3RA/8SA, were purified from ectopically expressing HEK cells in non-denaturing conditions by immobilisation in Flag-M2 affinity agarose beads and several washes containing 0.5% Triton X-100 and 1 M NaCl, and then eluted with a Flag peptide (Sigma-Aldrich). Recombinant GST-CHK1 was purchased from SinoBiological. Cleavage reaction was performed typically in 15 μL volume containing, 2 μg SPRTN, substrate (Flag-CHK1 or 0.2–1 μg GST-CHK1) and a 100 bp dsDNA oligonucleotide probe (Supplementary Table 2; 30:1 molar ratio SPRTN:dsDNA) in cleavage buffer (25 mM Tris, pH 7.4; 150 mM NaCl) at 37 °C for 4 h—o/n. The reaction was used straightaway for kinase assay or stopped by the addition of Laemmli buffer and boiling.

### In vitro CHK1 kinase assay

(A) Flag-CHK1 species (full length or mimicking cleavage products) were obtained from 10^8^ HEK cells previously transfected with expressing vectors and purified as above. (B) Recombinant GST-CHK1 was from commercial sources. For phosphorylation activity, purified Flag-CHK1 species were incubated (2 h) with 2 μg SPRTN, or alternatively 20–80 ng GST-CHK1 (either untreated or previously proteolysed by SPRTN) was incubated (30 min) with 200–500 ng cdc25A, at 37 °C in a kinase reaction buffer (10 mM HEPES, pH 7.4; 1 mM ATP; 10 mM MgCl_2_; 5 mM KCl; 25 mM β-glycerophosphate and phosphatase inhibitors) in a total volume of 25 μL. The reaction was stopped by the addition of 5× Laemmli buffer and boiling.

### Manipulation and analysis of Zebrafish embryos

Wild-type zebrafish (EK and AB strains) were kept under standardised conditions in a circulating water system (Tecniplast, Germany). Eggs were collected from natural matings. At the one to two cell stage, fertilized eggs were microinjected with a SPRTN or control morpholinos (sequences in Supplementary Information, Supplementary Table 2)^31^ and with in vitro synthesised, capped RNAs encoding different CHK1 variants fused to GFP, which were prepared from linearised plasmids using the mMessage mMachine SP6 Kit (Ambion). Alternatively, capped RNA encoding SPRTN variants were co-injected. Growth retardation was assessed at 9 h post fertilization (hpf; 90% epiboly stage under control conditions) using an upright brightfield M125 microscope equipped with a IC80 HD camera (both Leica). Immunofluorescence stainings were essentially prepared using a rabbit-anti-γH2Ax antibody (Genetex, 1:200 dilution) and imaged at a M205FA microscope equipped with a DFC365FX camera (both Leica)^66^.

### Mass spectrometry

Purified SPRTN was isolated by denaturing IP using Strep-Tactin beads (IBA), from 2.5 × 10^8^ HEK293 cells ectopically coexpressing SPRTN-E112A-SSH and either CHK1-WT or CHK1-S345A. SPRTN isolates were reduced with 5 mM DTT (Sigma-Aldrich; room temperature, 1 h), alkylated with 20 mM iodoacetamide (Sigma-Aldrich; room temperature, 1 h), and extracted using a double methanol/chloroform precipitation. Protein precipitates were resuspended in 6 M urea (with sonication), and diluted in a buffer containing 25 mM Tris–HCl (pH 8.5) and 1 mM EDTA until the urea concentration was <1 M. Protein was subsequently digested with rLysC (Promega) for overnight at 37 °C. Peptides were then desalted using a SOLA HRP SPE cartridge (Thermo Scientific Cat# 60109-001) and dried.

Dried tryptic peptides were reconstituted in 12 µL of LC-MS grade water containing 2% acetonitrile and 0.1% trifluoroacetic acid. Samples were subsequently analysed by nano-LC_MS/MS using a Dionex Ultimate 3000 UPLC coupled to a QExactive instrument (Thermo Scientific, Bremen, Germany)^67–69^. Peptides were online desalted on a PepMap C18 column (300 µm × 5 mm, 5 µm particle size, Thermo Fisher) for 1 min at a flow rate of 20 μL/min and separated on a directly coupled nEASY column (PepMap C18, 75 μm × 500 mm, 2 μm particle, Thermo Fisher) using a multistep gradient starting with 3 min at 2% acetonitrile in 5% DMSO with 0.1% formic acid (buffer B), followed by 60 min linear gradient up to 35% buffer B at 250 nL/min flow rate, 7 min linear gradient up to 99% buffer B and maintained at 99% buffer B for 5 min at a flow rate of 250 nL/min before reverting to 2% buffer B for 3 min prior to a last 4 min at 99% solvent B. Full MS scans were acquired over the *m*/*z* range of 380–1800 at a resolution of 70,000 at 200 *m*/*z* (AGC target of 3 × 10^6^ ions). MS/MS data was acquired in a data-dependent manner by selecting the 15 most abundant precursor ions for HCD fragmentation (CE of 28) and on MS/MS resolution of 17,500.

### Mass spectrometry data analysis

LC-MS/MS data was analysed by PEAKS (V7.5; Bioinformatics Solutions Inc., Waterloo, ON, Canada) using PEAKS database and PTM function. Data was searched against Human UniProtSwissProt database (downloaded on August 2016) enabling the decoy function whilst selecting LysC as enzyme (two missed cleavages; none non-specific cleavage), HCD as fragmentation source, mass tolerance of 10 ppm for precursor ions (MS data) and 0.05 Da for peptide fragment ions (MS/MS data); carbamidomethylation (C) as a fixed modification, and oxidation (M), deamidation (NQ) and phosphorylation (STY) as variable modifications. PEAKS-PTM results were filtered using a 1% FDR at peptide level. Extracted ion chromatogram (XIC) of peptides of interest was performed using Xcalibur Qual Browser (Thermo Scientific) enabling Gaussian peak smoothing at 3 points and a mass tolerance of precursor ion (MS1) of 4 ppm. Peaks areas were automatically calculated by the software. Mass spectrometry data analysis for phospho-sites detection performed by PEAKS software is contained in Supplementary Data 1.

### Statistics and reproducibility

Every experiment was reproduced at least three times, with similar results, except for the experiments shown in Figs. 2g and 7e, f, which were repeated twice. Unless otherwise stated, the statistical method used for comparison between experimental groups was unpaired two-tailed Student’s *t*-test carried out using GraphPad Prism. Statistical significance was expressed as a *P* value, where *P* < 0.05 was considered a statistically significant difference. Source Data contain all raw numbers used for statistical analysis.

### Reporting summary

Further information on research design is available in the Nature Research Reporting Summary linked to this article.

## Supplementary Information

Supplementary Information

Peer Review File

Supplementary Data 1

Description of Additional Supplementary Data 1

Reporting Summary

Source Data

## Data Availability

The Mass Spectrometry raw data reported in this paper have been deposited in the ProteomeXchange Consortium via the PRIDE^70^ partner repository with the dataset identifier PXD006741. The source data underlying Figs. 1c–k, 2b, c, f–h, 3a–c, e, f, 4b–f, h–k, 5a, c–g, i, j, 6a–f, i, j, 7a–f and 8a–c, and Supplementary Figs. S1d–h, S2a–b, S3b, S4a–c, S5a, b, e–i, S6a–e and S7a are provided as a Source Data file. All other data supporting the findings are available from the corresponding author upon reasonable request.
