# Supplementary material for: SPRTN protease and checkpoint kinase 1 cross-activation loop safeguards DNA replication
Source: Nat Commun. 2019 Jul 17;10:3142. doi: 10.1038/s41467-019-11095-y (PMC6637133; doi:10.1038/s41467-019-11095-y)
Supplement: Supplementary file 1 — Supplementary Information [file 41467_2019_11095_MOESM1_ESM.pdf]

Supplementary Information for

# SPRTN Protease and Checkpoint Kinase 1 Cross-Activation Loop Safeguards DNA Replication

Halder, Torrecilla et al.

**Supplementary Figure 1.** Related to main figure 1.

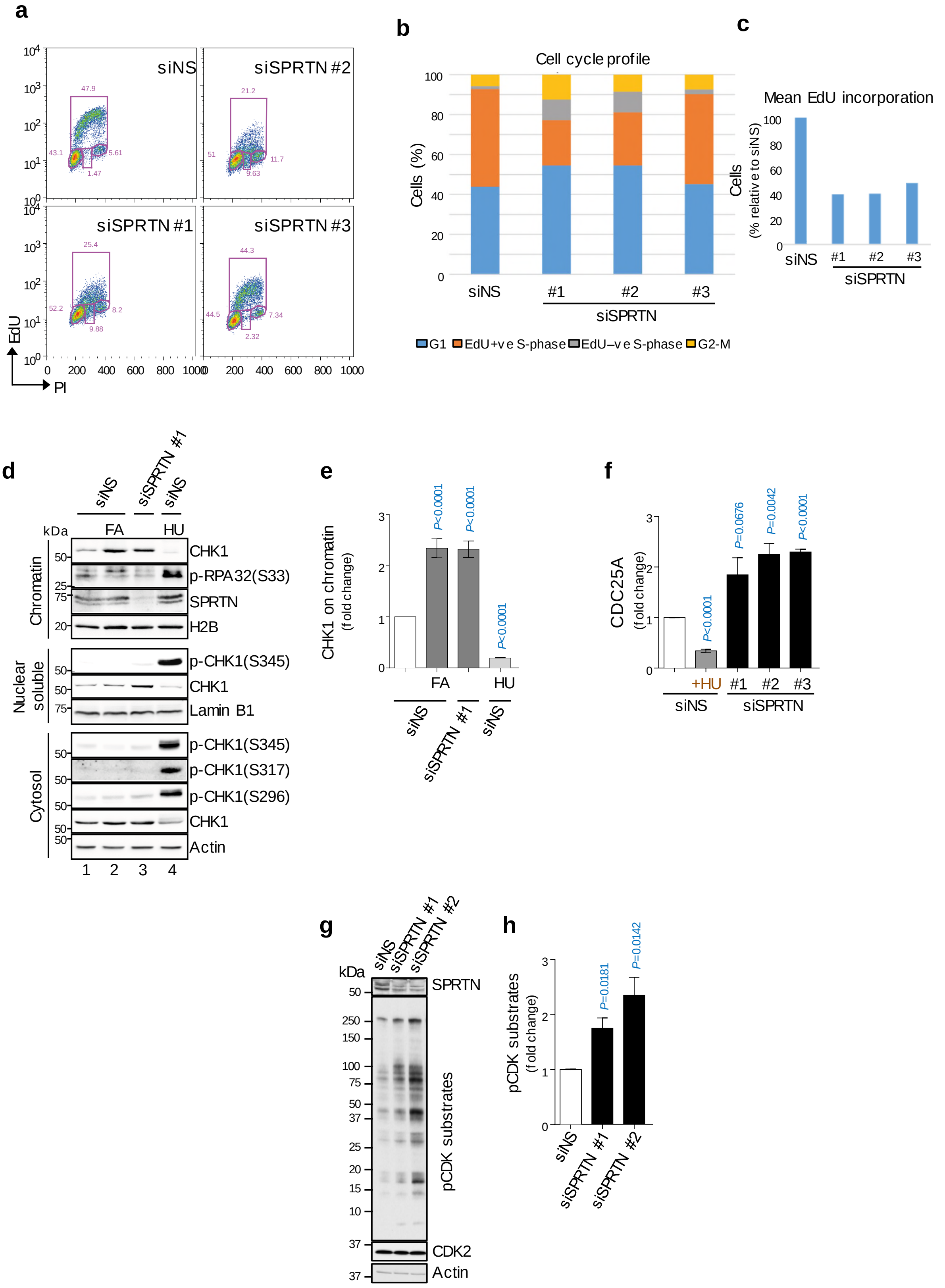

**Supplementary Figure 1. Analysis of cell cycle and CHK1 profiles in SPRTN-deficient cells.**

**a-c**, Cell cycle analysis of HEK293 cells depleted of SPRTN with three different siRNAs. **a**, propidium iodide (PI) profiling. **b**, cell cycle profile from quantification of **a**. **c**, EdU incorporation in cells in **a**. Data are representative of three replicates.

**d-e**, CHK1 was enriched on chromatin fraction (see also main Fig. 4a, b) in HEK293 cells depleted of SPRTN or treated with formaldehyde (FA; 50 μM, 1 h) and its phosphorylation was diminished. Cells were subjected to cellular fractionation to independently analyse chromatin fraction (upper panel of immunoblots) and phosphorylation in nuclear soluble and cytosolic fractions (medium and lower panels, respectively). **e** shows quantification of CHK1 on chromatin. Mean ± SEM; n=3 replicates, two-tailed Student's *t*-test.

**f**, Stabilization of Cdc25A total protein level in SPRTN-depleted HEK293 cells. Data are quantifications of immunoblots from main Fig. 1f. Mean ± SEM; n=3 replicates, two-tailed Student's *t*-test.

**g-h,** Enhancement of phospho-CDK substrates in SPRTN-depleted HEK293 cells. Data display representative immunoblots of whole cell lysates using a specific antibody that recognizes phosphorylated CDK substrates (**g**), and its quantification normalised to actin (**h**). Mean ± SD; n=3 replicates, two-tailed Student's *t*-test.

**Source data for Supplementary Figure 1d-h are provided as a Source Data file.**

Supplementary Figure 2. Related to main figure 2.

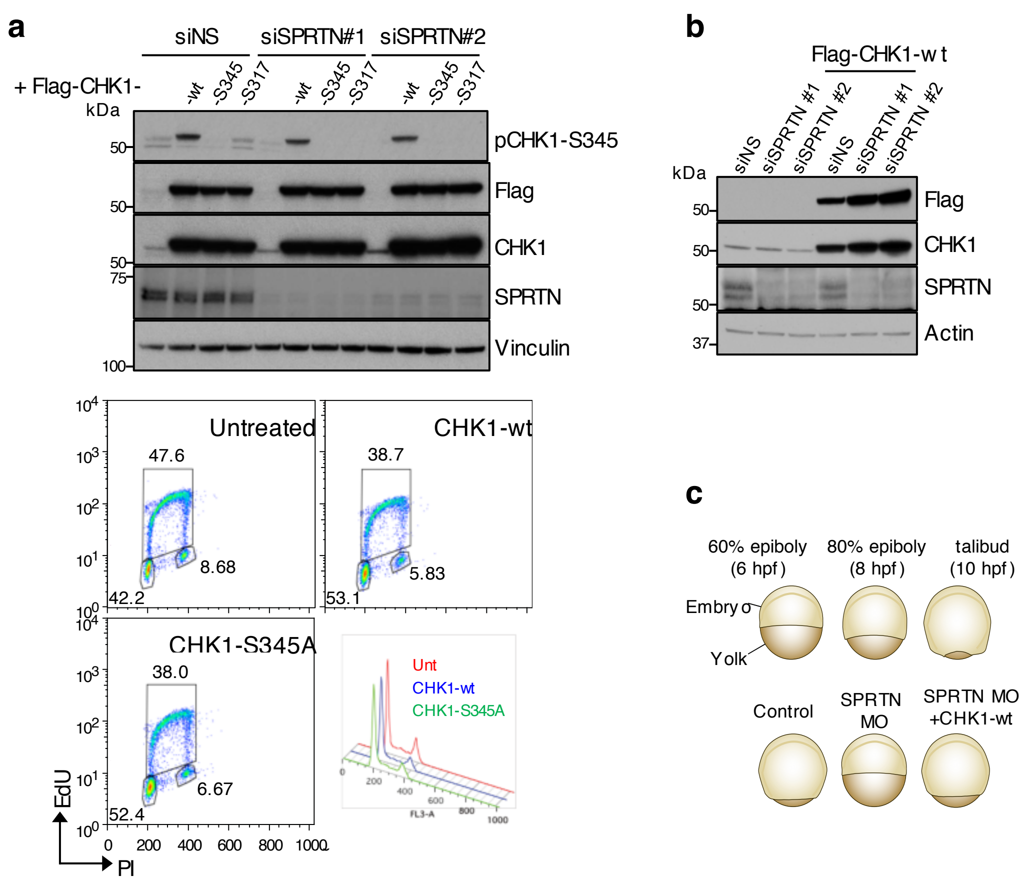


**Figure S2. Ectopic expression of CHK1.**

**a**, Immunoblots showing the expression and phosphorylation degree of different CHK1 variants overexpressed in HEK293 cells (upper panel), and cell cycle profile of HEK293 cells as indicated (lower panel). Related to main Fig. 2b.

**b**, Immunoblots showing the efficiency of SPRTN depletion alone or combined with the expression of CHK1-wt. Related to main Fig 2c.

**c**, Graphical illustration of embryos developing normally after 6, 8 and 10 hours post fertilization (hpf; upper row), and of embryos at 10 hpf at the indicated conditions (lower row) as observed in Fig. 2d.

**Source data for Supplementary Figure S2a-b are provided as a Source Data file.**

**Supplementary Figure 3.** Related to main figure 3.

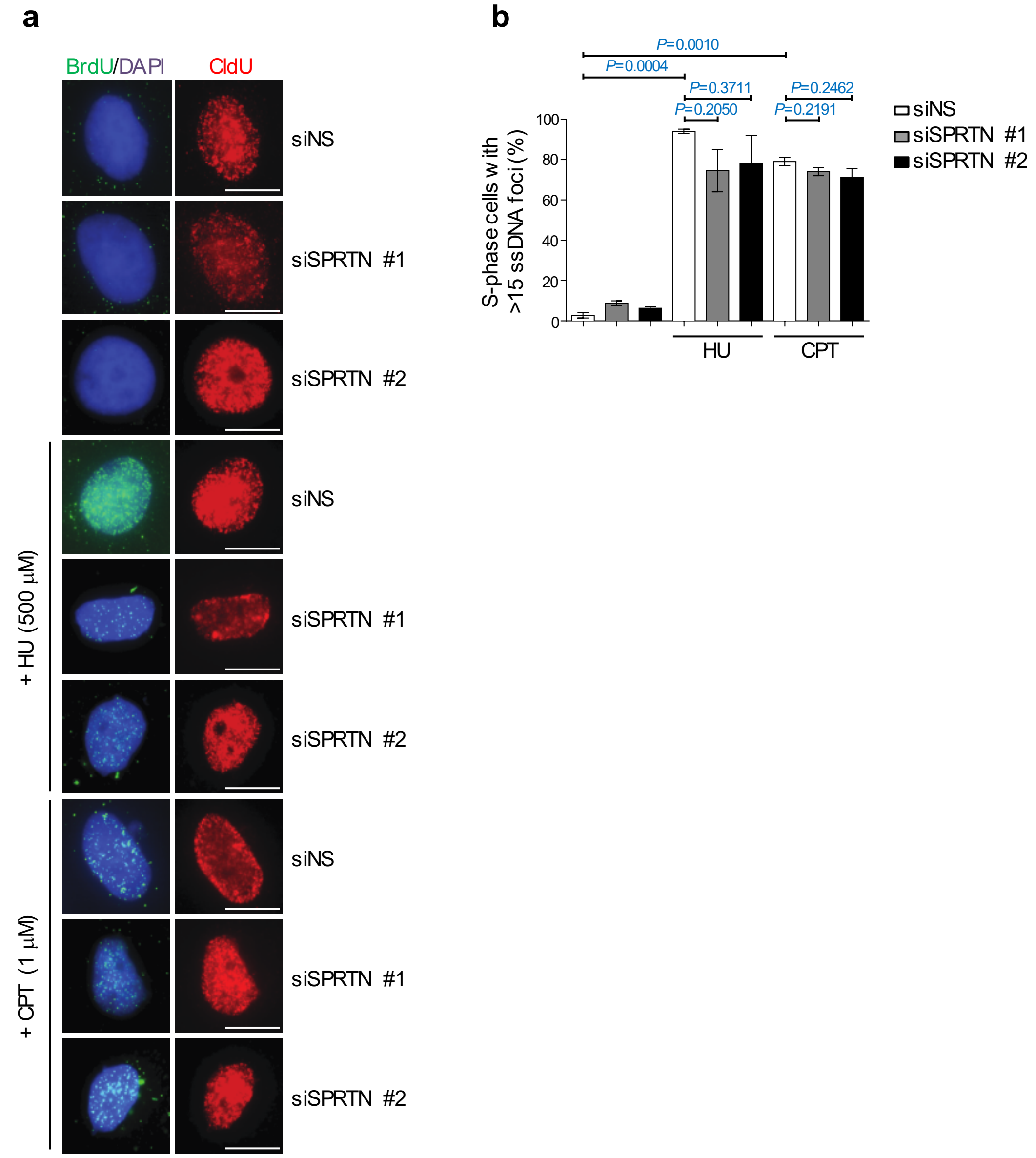


**Supplementary Figure 3. SPRTN-deficient cells retain the ability to form ssDNA after hydroxyurea induced replication stress or camptothecin inducd DNA double strand formation.**

**a,** Representative immunofluorescent microscopy images of BrdU foci (marker for ssDNA) in S-phase (marked by CldU positive cells) HeLa cells treated with either siNS or siSPRTN and hydroxyurea (HU) or camptothecin (CPT). Scale bar = 10µm.

**b**, quantification of ssDNA in **a**. >70 S-phase cells were scored per condition per experiments. Mean ± SEM, n=2 experimental replicates, two-tailed Student's *t*-test.

**Source data for Supplementary Figure 3b are provided as a Source Data file.**

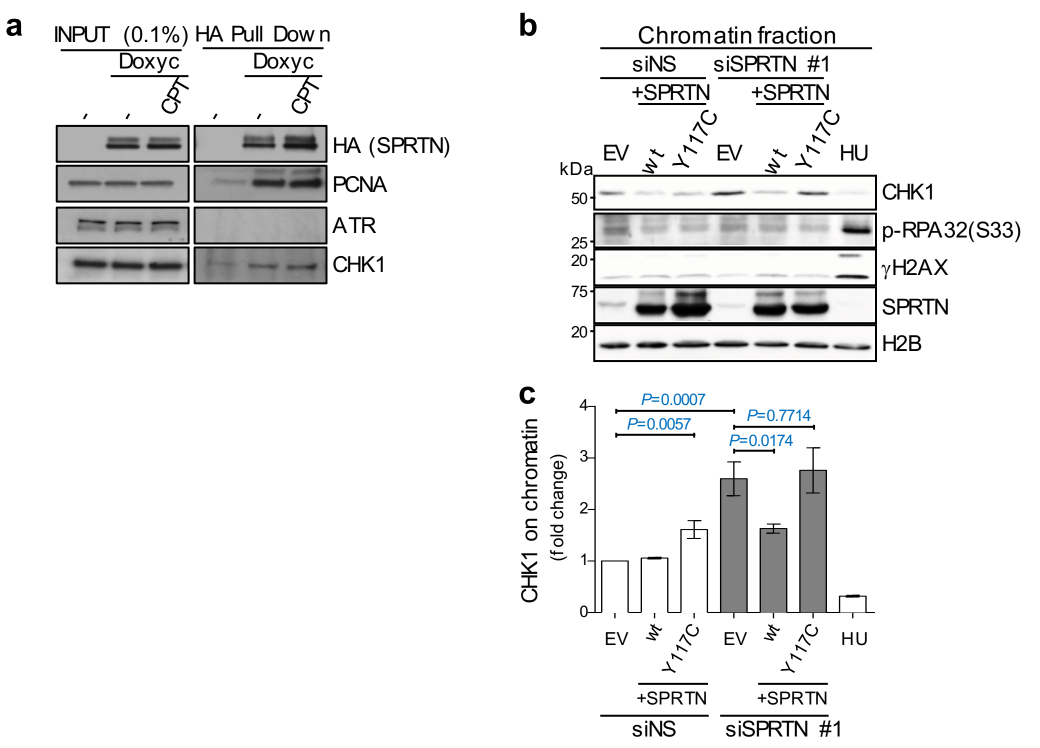


**Supplementary Figure 4. SPRTN protease evicts CHK1 kinase from chromatin**

**a,** HA pull-down assay to detect interactions of SPRTN-HA in stable Flp-In HEK293 T-REx cells. SPRTN interacted with CHK1, but *in vivo* interaction between SPRTN and ATR could not be detected. Immunoblots represent three replicates. Doxyc: doxycycline (1 µg/ml, 16 h); CPT: camptothecin (100 nM).

**b**, Immunoblot analysis of chromatin fractions from control or SPRTN-depleted HEK293 cells co-transfected with empty vector (EV), SPRTN-WT or SPRTN-Y117C (patient variant with decreased protease activity), showing CHK1 accumulation in chromatin in SPRTN-deficient cells. pRPA2 and DNA damage (γH2AX) are shown as markers for DNA damage. Hydroxyurea (HU, 1 mM, 1 h) was used as a positive control.

**c,** Quantification of CHK1 in **b**, normalised to histone H2B. Mean ± SEM, n = 5 experiments, two-tailed Student´s *t*-test.

**Source data for Supplementary Figure 4a-c are provided as a Source Data file.**

**Supplementary Figure 5.** Related to main figure 5.

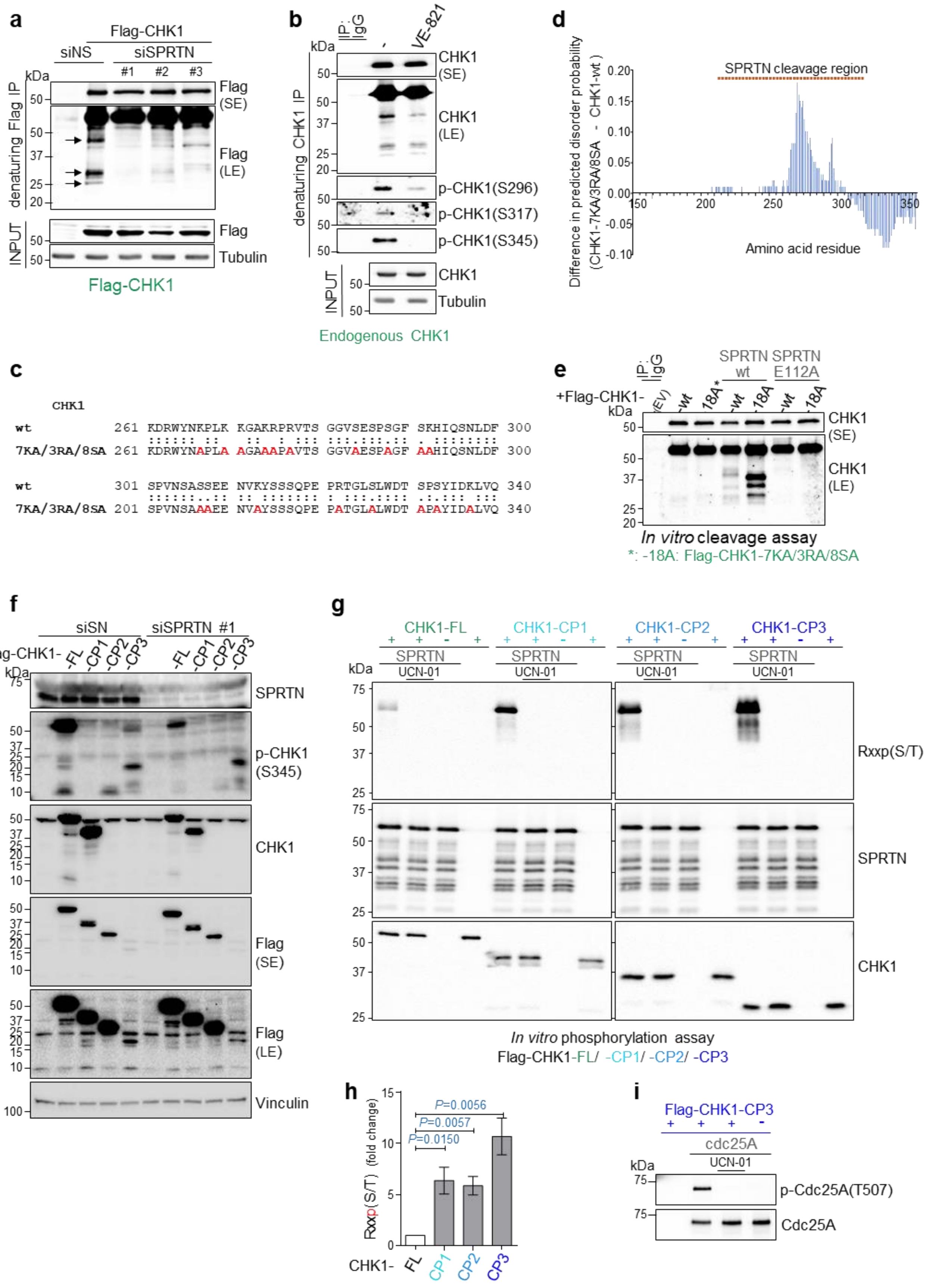

**Supplementary Figure 5. SPRTN cleaves CHK1 and releases kinase-active CHK1 fragments.**

**a,** SPRTN protease generates CHK1 fragments. Whole cell extracts from HEK293 cells ectopically expressing Flag-CHK1 were SDS-denatured and CHK1 was then immuno-purified using anti Flag-beads. Representative immunoblots from three replicates.

**b**, ATR is involved in CHK1 cleavage. Endogenous CHK1 from HEK293 lysates treated or not with the ATR inhibitor VE-821 was immuno-purified using an antibody recognising a CHK1 N-terminal epitope. Representative immunoblots from three experimental replicates.

**c**, Sequence alignment of CHK1-wt and the CHK1-7KA/3RA/8SA mutant showing mutated sites in red colour. The mutated residues were chosen for their location by the estimated SPRTN cleavage site.

**d**, Predicted disorder probability in the region of SPRTN cleavage is increased in the CHK1-7KA/3RA/8SA mutant. Positive or negative Y-axis values represent a higher disorder probability in CHK1-7KA/3RA/8SA or in Chk1-wt, respectively. Disordered probability for each residue was predicted using SCRATCH protein predictor (http://scratch.proteomics.ics.uci.edu/).

**e**, SPRTN cleaves the CHK1-7KA/3RA/8SA mutant more efficiently than CHK1-*wt in vitro*. Flag-CHK1 variants were purified from HEK293 cells and incubated with SPRTN-wt or SPRTN-E112A. An antibody against a CHK1 N-terminal epitope was used. Loading was adjusted to equalise full length CHK1. Representative immunoblots from three replicates.

**f**, Expression of Flag-CHK1 variants in control and SPRTN-depleted cells (Flag tag located at the N-terminus). Anti-CHK1 antibody against a C-terminally located epitope could not detect CHK1-CP2 and CHK1-CP3 due to loss of epitope. Related to main Fig. 5f.

**g-h**, *In vitro* phosphorylation assay (g) and quantification (h) showing that all three CHK1-CPs are kinase active and phosphorylate SPRTN with higher specific activities than CHK1-FL. Flag-CHK1 variants were purified from HEK293 cells; same amounts of protein were incubated with SPRTN protein at 37°C for 10 min. CHK1-mediated phosphorylation was detected with a specific antibody recognising the sequence R-x-x-p(S/T), a CHK1-phosphorylated substrate consensus sequence.

**i**, CHK1-CP3 is also kinase active towards the well-characterised CHK1 substrate Cdc25A (0.5 μg, 2h) *in vitro*. Representative of three replicates.

**Source data for Supplementary Figure 5a, b, e-i are provided as a Source Data file.**

**Supplementary Figure 6.** Related to main figure 5.

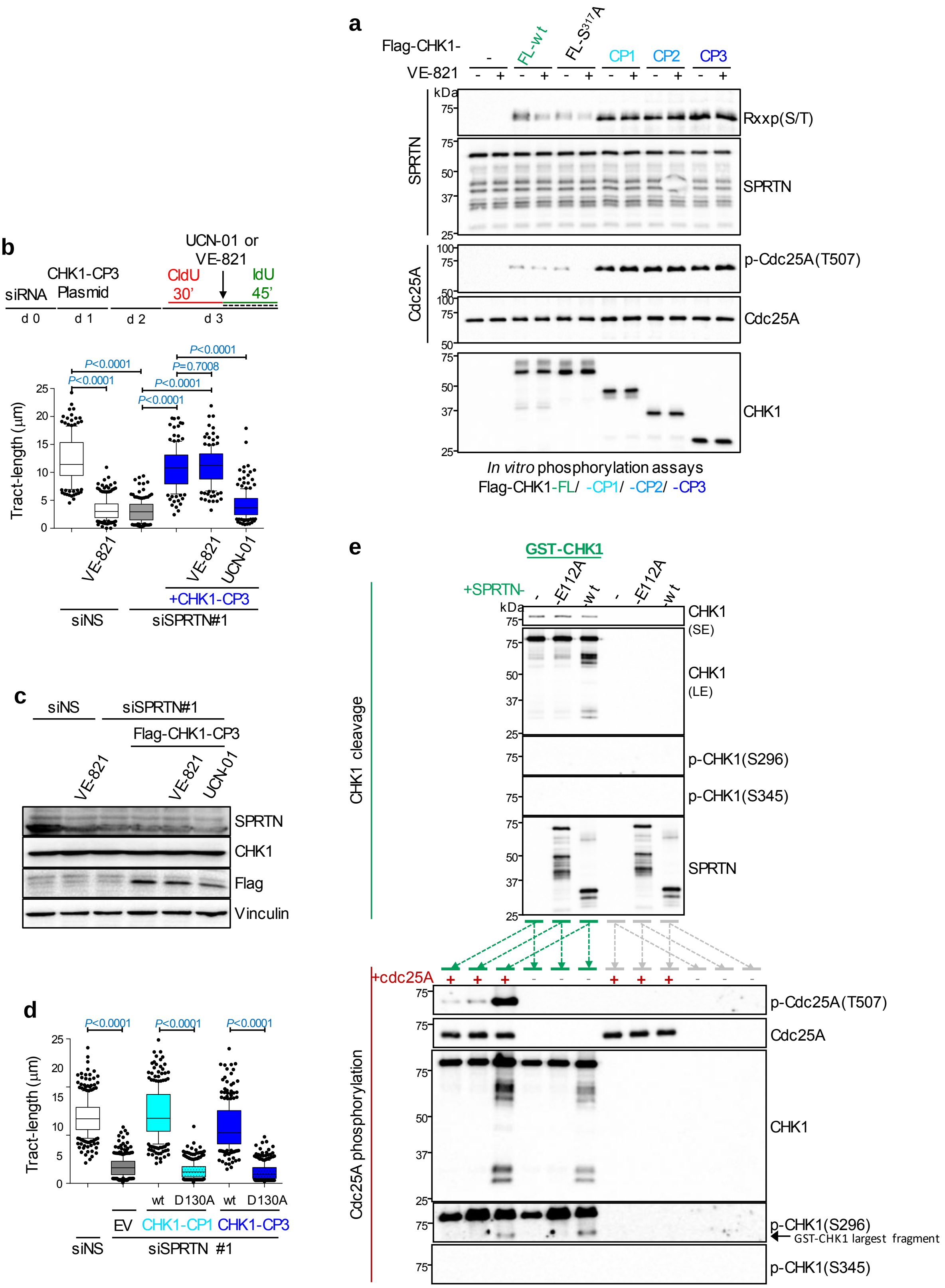

**Supplementary Figure 6. Analysis of the activity of SPRTN-released CHK1-fragments.**

**a**, The kinase activity of CHK1-CPs is not affected by ATR. Flag-CHK1 species were purified from HEK293 cells pre-treated or not with the ATR inhibitor VE-821 (5 μM, 10 h). Equal amounts of CHK1 were used for *in vitro* kinase assays on SPRTN or Cdc25A. n = 2.

**b**, Analysis of DNA replication fork velocity by DNA fiber assay. The ATR inhibitor VE-821 (5 μM) or the CHK1 inhibitor UCN-01 (300 nM) severely slow DNA replication fork velocity in control cells (siNS). Related to main Fig. 5f. Ectopic expression of the smallest N-terminal CHK1 fragment (CP3) rescues DNA replication fork velocity in SPRTN-depleted cells (siSPRTN). The rescue effect of CHK1-CP3 fragment on DNA replication fork velocity in SPRTN-depleted cells is CHK1-kinase dependent (sensitive to UCN-01) but ATR kinase-independent (resistant to VE-821). Upper diagram shows the experimental strategy (d: day). Graph below shows the data for replication fork velocity (mean ± 25-75 percentile range (box) and 10-90 percentile range (whiskers)). > 100 DNA fibers were analysed per condition and experiment; n=3 experiments, two-tailed Student´s *t*-test.

**c**, Immunoblot analysis for **b** showing the efficiency of SPRTN depletion and the expression of Flag-CHK1-CP3 in the presence of the CHK1 inhibitor UCN-01 or the ATR inhibitor VE-821.

**d**, The active kinase domain of the N-terminal CHK1 fragments (CP1 and CP3) is required for the rescue of DNA replication defects in SPRTN-depleted HEK293 cells. Related to main Fig. 5f. D130A: kinase dead CHK1 variants with a mutation in Aspartic acid (D) 130 to Alanine (A) located in the catalytic domain. Data shown and statistics as in Supplementary Figure 6b.

**e**, SPRTN activates CHK1 by cleavage and release of kinase-active fragments. Related to main Fig. 5h, i. Top panel: GST-CHK1 was cleaved by SPRTN-wt generating fragments of different sizes. Bottom panel: Only the largest of the CHK1 fragment exhibited phosphorylation at residue Ser296. Experiment was repeated 3 times with similar results.

**Source data for Supplementary Figure 6a-e are provided as a Source Data file.**

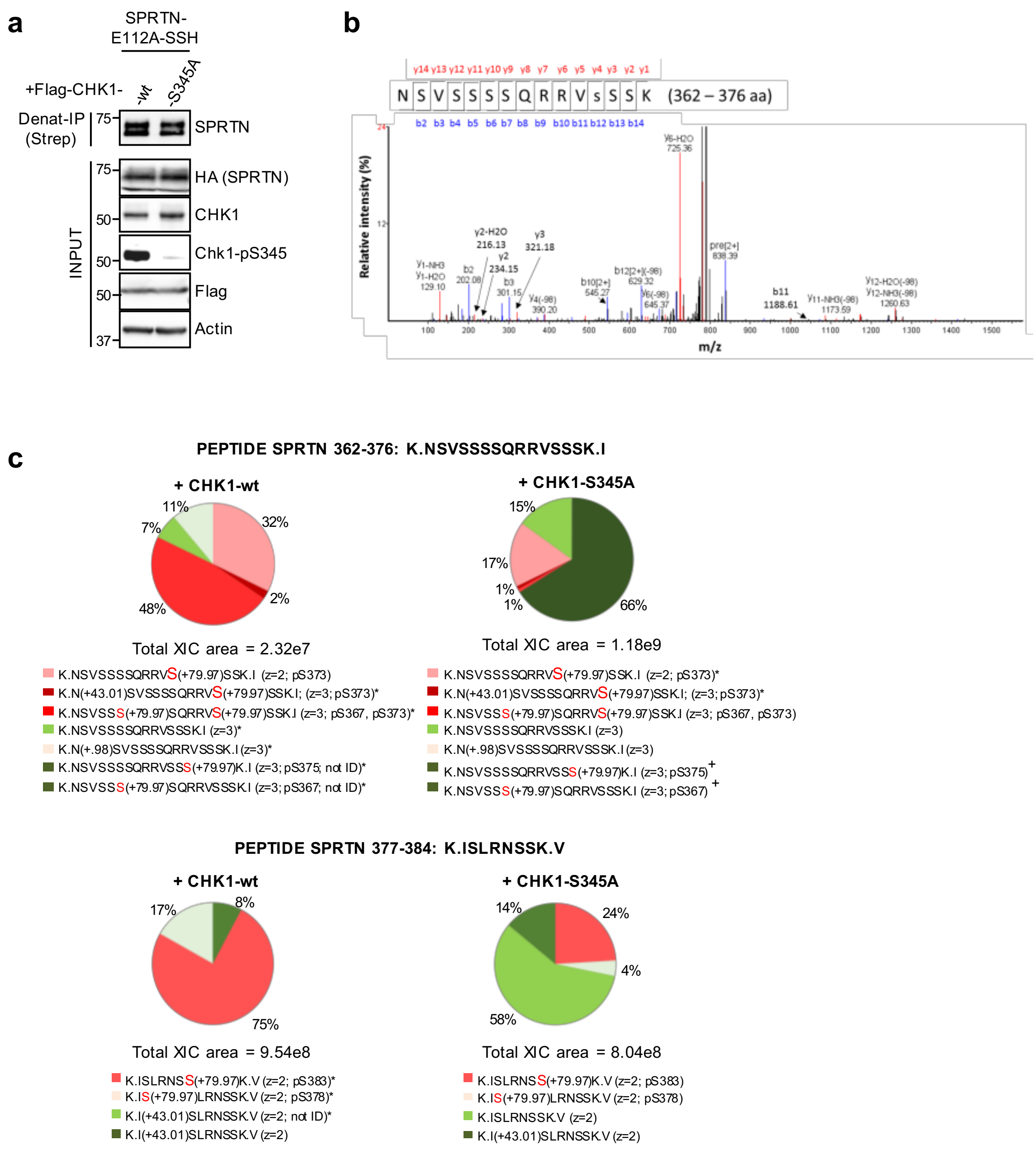


**Supplementary Figure 7. Identification of CHK1 phospho-targets in SPRTN by Mass-Spectrometry.**

**a**, Samples for Mass-Spectrometry analysis were prepared by co-expression of the protease-dead mutant SPRTN-E112A (to prevent autocleavage) tagged with Strep-Strep-HA with either CHK1-wt or the CHK1-S345A mutant, followed by denaturing-IP isolation of SPRTN. Related to main Fig. 6g.

**b**, Example of MS/MS fragmentation spectra (PEAKS; intensity up to 24%) of SPRTN peptide 362-376 (K.NSVSSSSQRRVSSSK.I) detected in the CHK1-WT overexpression sample, showing a phosphorylation in amino acid S373. Arrows point to the b and y ions which strongly support that the phosphorylation site is on S373 (p-site localization score of 8.18).

**c**, Extracted ion chromatograms (XIC; 4ppm) for SPRTN 362-376 peptide (top panel) and for SPRTN 377–384 peptide (bottom panel) containing amino acids S373, 374 and S383, respectively, comparing peptides species detected when CHK1-wt (left) or CHK1-S345 (right) was overexpressed. Each red tone corresponds to different peptides species containing potential phosphorylation at S373, 374 or S383, whereas green tones correspond to non-phosphorylated peptides. PTMs were automatically assigned by PEAKS software. Pie charts areas represent peptide intensities. *: Peptide identification performed by matching m/z and retention time from existent data. Not ID = not identified. + co-eluting peptides with almost identical m/z and retention time but with different p-site assignment. Related to main Fig. 6h.

**Source data for Supplementary Figure 7a are provided as a Source Data file. Source Mass Spectrometry raw data is available (see Methods).**

## Supplementary Table 1 - Resources used in this study

This table lists main chemicals, antibodies, bacterial strains, commercial assays, experimental models (cell lines and organisms), plasmids, software and algorithms used in this study.

| Resource | Source | Catalog # | Notes |
|---|---|---|---|
| **Experimental models:** | | | |
| **Human cell lines** | | | |
| Human: embryonic kidney HEK293 cells (female) | ATCC | CRL-1573, RRID:CVCL_0045 | |
| Human: osteosarcoma U-2 OS cells (female) | ATCC | HTB-96, RRID:CVCL_0042 | |
| Human: cervical carcinoma HeLa cells (female) | ATCC | CCL-2, RRID:CVCL_0030 | |
| Human: CRISPR delta-SPRTN HeLa cells | Ramadan lab: Vaz et al., 2016 | N/A | |
| Human: HEK293-Flp-In TRex SPRTN-wt-cSSH | Ramadan lab: Vaz et al., 2016 | N/A | |
| Human: HEK293-Flp-In TRex SPRTN-Y117A-cSSH | Ramadan lab: Vaz et al., 2016 | N/A | |
| Human: T24 bladder cancer cell line | Ramadan lab: Vaz et al., 2016 | N/A | |
| **Experimental models: organisms** | | | |
| Zebrafish: AB strain | Philipp lab | N/A | |
| Zebrafish: EK strain | Philipp lab | N/A | |
| **Bacterial strains** | | | |
| *Escherichia coli* DH5a | Invitrogen | 18265-017 | Chemically competent |
| *Escherichia coli* Rosetta 2 (DE3) | Novagen | 71405-3 | Chemically competent |
| **Chemicals, Peptides, and Recombinant Proteins** | | | |
| DMEM | Sigma-Aldrich | D6429 | |
| FBS | Gibco | 10270 | |
| Penicillin / Streptomycin | Sigma-Aldrich | P4333 | |
| Mycoalert mycoplasma detection kit | Lonza | LT07-218 | |
| Precision Plus Protein Dual Color Standards | Bio-Rad | 1610394 | |
| Nitrocellulose membrane | Sigma-Aldrich | GE10600008 | |
| PVDF membrane | Bio-Rad | 1620177 | |
| Prolong Gold antifade | ThermoFisher | P36930 | |
| Hydroxyurea | Sigma-Aldrich | H8627 | |
| Camptothecin | Calbiochem | 208925 | |
| Formaldehyde | Fisher Scientific | F/1501/PB08 | |
| 5-Chloro-2' deoxyuridine (CldU) | Sigma-Aldrich | C6891 | |
| 5-Iodo-2'-deoxyuridine (IdU) | Sigma-Aldrich | I7125 | |
| 5-Ethynyl-2'-deoxyuridine (EdU) | Sigma-Aldrich | T511285 | |
| 5-Bromo-2'-deoxyuridine (BrdU) | Sigma-Aldrich | B9285 | |
| UCN-01, CHK1 inhibitor | Sigma-Aldrich | U6508 | |
| NSC 663284, cdc25 phosphatase inhibitor | Santa Cruz | sc-202987 | |
| Roscovitine, CDK inhibitor | Sigma-Aldrich | R7772 | |
| VE-821, ATR inhibitor | Sigma-Aldrich | SML1415 | |
| Doxycycline | Panreac | A2951,0025 | |
| Accuprime Pfx DNA polymerase | Invitrogen | 12344-024 | |
| FuGene HD | Promega | E2311 | |

| | | | |
|---|---|---|---|
| Lipofectamine siRNAMax | Invitrogen | 13778-150 | |
| Polyethylenimine | Sigma-Aldrich | 408727 | |
| Benzonase, nuclease | Millipore | 71205 | |
| rLysC, Mass Spec Grade | Promega | V1671 | |
| Strep-Tactin Sepharose | IBA | 2-1201-010 | |
| Anti-Flag M2 affinity gel | Sigma-Aldrich | A2220 | |
| Flag peptide | Sigma-Aldrich | F3290 | |
| Streptavidin-coupled agarose beads | Merck Millipore | 69203 | IPOND |
| SPRTN-wt protein | Ramadan lab: Vaz et al., 2016 | N/A | |
| SPRTN-E112A protein | Ramadan lab: Vaz et al., 2016 | N/A | |
| GST-CHK1 protein | SinoBiological | 10539-H09B | |
| Cdc25A protein | Raybiotech | 230-00645-50 | CHK1 kinase assays |
| Proteinase K | Invitrogen | 25530-015 | |
| **Recombinant DNA** | | | |
| pCDNA5-FRT/TO-cSSH | Ramadan lab: Vaz et al., 2016 | N/A | |
| pCDNA5-FRT/TO-SPRTN-wt-cSSH | Ramadan lab: Vaz et al., 2016 | N/A | |
| pCDNA5-FRT/TO-SPRTN-Y117C-cSSH | Ramadan lab: Vaz et al., 2016 | N/A | |
| pCDNA5-FRT/TO-SPRTN-E112A-cSSH | Ramadan lab: Vaz et al., 2016 | N/A | |
| pCDNA5-FRT/TO-SPRTN-S373A-cSSH | This paper | N/A | |
| pCDNA5-FRT/TO-SPRTN-S374A-cSSH | This paper | N/A | |
| pCDNA5-FRT/TO-SPRTN-S383A-cSSH | This paper | N/A | |
| pCDNA5-FRT/TO-SPRTN-S373A/S374A-cSSH | This paper | N/A | |
| pCDNA5-FRT/TO-SPRTN-S373A/S374A/S383A-cSSH | This paper | N/A | |
| pCDNA5-FRT/TO-SPRTN-S373E-cSSH | This paper | N/A | |
| pCDNA5-FRT/TO-SPRTN-S374E-cSSH | This paper | N/A | |
| pCDNA5-FRT/TO-SPRTN-S383E-cSSH | This paper | N/A | |
| pCDNA5-FRT/TO-SPRTN-S373E/S383E-cSSH | This paper | N/A | |
| pCINeo/CMV-Flag-Chk1-WT | Smits et al., 2005 | N/A | |
| pCINeo/CMV-Flag-Chk1-S345A | This paper | N/A | |
| pCINeo/CMV-Flag-Chk1-S317A | This paper | N/A | |
| pCINeo/CMV-Flag-Chk1_1-237 | This paper | N/A | Expression of CHK1-CP1 |
| pCINeo/CMV-Flag-Chk1_1-293 | This paper | N/A | Expression of CHK1-CP2 |
| pCINeo/CMV-Flag-Chk1_1-338 | This paper | N/A | Expression of CHK1-CP3 |
| pCS2-Chk1-wt-GFP | This paper | N/A | |
| pCS2-Chk1-S345A-GFP | This paper | N/A | |
| pCS2-Chk1-S317A-GFP | This paper | N/A | |
| pCS2-Chk1_1-237-GFP | This paper | N/A | |
| pCS2-SPRTN-wt | Ramadan lab: Lessel et al., 2014 | N/A | |
| pCS2-SPRTN-S373A/S374A | This paper | N/A | |
| pCS2-SPRTN-S383A | This paper | N/A | |
| pCS2-SPRTN-S373A/S374A/S383A | This paper | N/A | |
| pCS2-SPRTN-S373E | This paper | N/A | |
| pCS2-SPRTN-S383E | This paper | N/A | |
| pCS2-SPRTN-S373E/S383E | This paper | N/A | |
| pNIC-ZB-SPRTN-wt | Ramadan lab: Vaz et al., 2016 | N/A | |

| | | | |
|---|---|---|---|
| pNIC-ZB-SPRTN-E112A | Ramadan lab: Vaz et al., 2016 | N/A | |
| **Antibodies** | | | |
| CHK1 (Mouse monoclonal) | Cell Signaling Technology | 2360; RRID:AB_2080320 | |
| CHK1 [G-4] (Mouse monoclonal) | Santa Cruz Biotechnology | sc-8408; RRID:AB_627257 | |
| CHK1 [E250] (Rabbit monoclonal) | Abcam | ab32531; RRID:AB_726821 | |
| phospho-CHK1 (Ser296) (Rabbit monoclonal) | Abcam | ab79758; RRID:AB_2244917 | |
| phospho-CHK1 (Ser317) (Rabbit polyclonal) | Cell Signaling Technology | 2344S; RRID:AB_331488 | |
| phospho-CHK1 (Ser345) (Rabbit monoclonal) | Cell Signaling Technology | 2348L; RRID:AB_2080326 | |
| CHK2 (Rabbit polyclonal) | Cell Signaling Technology | 2662; RRID:AB_2080793 | |
| phospho-CHK2 (Thr68) (Rabbit polyclonal) | Cell Signaling Technology | 2661S; RRID:AB_331479 | |
| Phospho-gammaH2AX (Ser139) (Rabbit polyclonal) | Novus Biologicals | NB100-2280; RRID:AB_10000580 | |
| Phospho-gammaH2AX (Ser139) | Genetex | GTX127342 | |
| HA [clone 3F10] (Rat monoclonal) | Roche | 3F10; RRID:AB_2314622 | |
| PCNA (Mouse monoclonal) | Abcam | ab29; RRID:AB_303394 | |
| RPA32 (Rabbit polyclonal) | Bethyl Laboratories | A300-244A; RRID:AB_185548 | |
| phospho-RPA32 (Ser4/8) (Rabbit polyclonal) | Bethyl Laboratories | A300-245A; RRID:AB_210547 | |
| Phospho-RPA32 (Ser33) (Rabbit polyclonal) | Bethyl Laboratories | IHC-00421; RRID:AB_1659839 | |
| Vinculin (Mouse monoclonal) | Bethyl Laboratories | ab18058; RRID:AB_444215 | |
| Flag (Rabbit polyclonal) | Sigma-Aldrich | F7425; RRID:AB_439687 | |
| Flag [clone M2] (Mouse monoclonal) | Sigma-Aldrich | F1804; RRID:AB_262044 | |
| CDC25A [clone F-6] (Mouse monoclonal) | Santa Cruz Biotechnology | sc-7389; RRID:AB_627226 | |
| phospho-CDC25A (Thr507) (rabbit polyclonal) | Abgen | AP3051a; RRID: AB_2075420 | |
| phospho-CDK substrate motif [(K/H)pSP] (Rabbit monoclonal) | Cell Signaling Technology | 9477s; RRID:AB_2714143 | |
| CDK2 (Rabbit polyclonal) | Abcam | ab6538; RRID:AB_305552 | |
| phospho-PPP1A (Thr320) (Rabbit monoclonal) | Abcam | ab62334; RRID:AB_956236 | |
| PPP1A [clone [1C11-C10-H5-E9] (Mouse monoclonal) | Abcam | ab150782 | |
| phospho Rxxp(S/T) substrate (Rabbit monoclonal) | Cell Signaling Technology | 6950S; RRID:AB_10827652 | |
| SPRTN N-terminal (Rabbit polyclonal) | In house; Lessel *et.al*, 2014 | N/A | |
| SPRTN C-terminal (Rabbit polyclonal) | In house; Lessel *et.al*, 2014 | N/A | |
| ATM (Mouse monoclonal) | Sigma-Aldrich | A1106; RRID:AB_796190 | |
| phospho-ATM (Ser1981) (Rabbit monoclonal) | Abcam | ab81292; RRID:AB_1640207 | |
| beta-Actin (Mouse monoclonal) | Abcam | ab6276; RRID:AB_2223210 | |
| H2B (Mouse monoclonal) | Cell Signaling Technology | 2934S; RRID:AB_2295301 | |
| MCM3 [clone E-7] (Mouse monoclonal) | Santa Cruz Biotechnology | sc-365616; RRID:AB_10846721 | |
| Histone H3 (Rabbit polyclonal) | Abcam | ab1791; RRID:AB_302613 | |
| ds-DNA (Mouse monoclonal) | Abcam | ab27156; RRID:AB_470907 | |
| Cyclin E [clone HE12] (Mouse monoclonal) | Millipore | 05-363; RRID:AB_2071085 | |
| Cyclin A [clone H-432] (Rabbit polyclonal) | Santa Cruz Biotechnology | sc-751; RRID:AB_631329 | |
| Lamin B1 (Rabbit polyclonal) | Thermo Fisher Scientific | PA5-19468; RRID:AB_10985414 | |
| Rabbit IgG, HRP-conjugated (goat polyclonal) | Sigma-Aldrich | A9169; RRID:AB_258434 | 1:60,000 |
| Mouse IgG, HRP-conjugated (rabbit polyclonal) | Sigma-Aldrich | A9044; RRID:AB_258431 | 1:60,000 |
| Rat IgG, HRP-conjugated (goat polyclonal) | Bio-Rad | 5204-2504; RRID:AB_619913 | 1:10,000 |
| BrdU [clone BU1/75(ICR1)] (rat monoclonal) | Abcam | ab6326 RRID:AB_305426 | DNA fibres |
| BrdU [clone B44] (mouse monoclonal) | BD Biosciences | 347580 RRID:AB_400326 | DNA fibres |

| | | | |
|---|---|---|---|
| Rat IgG, Cy3-AffinityPureF(ab')2 Fragment (donkey polyclonal) | Jackson Immuno Research | 712-166-153 RRID:AB_2340669 | DNA fibres |
| Mouse IgG, Alexa Fluor 488 (goat polyclonal) | Molecular Probes | A-11001; RRID:AB_2534069 | DNA fibres |
| dsDNA (mouse monoclonal) | Abcam | ab27156; RRID AB_470907 | |
| **Commercial assays** | | | |
| Clarity™ Western ECL Substrate | Bio-Rad | 1705061 | |
| SuperSignal™ West Femto Maximum Sensitivity Substrate | ThermoFisher Scientific | 34095 | |
| SuperSignal™ West Pico PLUS Chemiluminescent Substrate | ThermoFisher Scientific | 34580 | |
| Click-iT™ EdU Alexa Fluor™ 647 Imaging Kit | ThermoFisher Scientific | C10340 | |
| ProteoSilver Plus Silver Stain Kit | Sigma-Aldrich | PROTSIL2 | |
| **Deposited data** | | | |
| Raw mass spectrometry data | ProteomeXchange Consortium via the PRIDE partner repository | ProteomeXchange code: PXD006741 | |
| **Software and algorithms** | | | |
| PEAKS V7.5 | Bioinformatics Solutions Inc | http://www.bioinfor.com/peaks-studio/ | |
| Xcalibur 3.1 (Qual Browser) | Thermo Scientific | https://www.thermofisher.com/order/catalog/product/OPTON-30487 | |
| Graphpad Prism v6.01 | Graphpad Software | http://www.graphpad.com/ | |
| ImageLab v5.2.1 | Bio-Rad Laboratories | http://www.bio-rad.com/en-ch/product/image-lab-software | |
| ImageJ 1.48v | Wayne Rasband (NIH) | https://imagej.nih.gov/ij/ | |

**Supplementary Table 2** - Oligonucleotides used in this study (siRNA, morpholino, subcloning primers, site-directed mutagenesis primers)

| Name | Oligonucleotide sequence | Source | Purpose |
|---|---|---|---|
| **siRNAs** | | | |
| siRNA-SPRTN #1 | 5'-GUCAGGAAGUUCUGGUUAA-3' | Ramadan lab: Vaz et al., 2016 | siRNA targeting SPRTN protein |
| siRNA-SPRTN #2 | 5'-CACGAUGAGGUGGAUGAGUAU-3' | Ramadan lab: Vaz et al., 2016 | siRNA targeting SPRTN protein |
| siRNA-SPRTN #3 | 5'-AGCCAAUAUAACGGUAUACCA-3' | Ramadan lab: Vaz et al., 2016 | siRNA targeting SPRTN protein |
| siNS (control) | 5'-CGUACGCGGAAUACUUCGA-3' | This study | siRNA targeting luciferase |
| **Morpholinos** | | | |
| SPRTN Morpholino | 5'- AAAGTCTTCATCCTCCATCATCTTC-3' | This study | Morpholino targeting SPRTN protein |
| Control morpholino | 5'- AAACTCTTGATCGTCCATGATGTTC-3' | | |
| **Primers for subcloning** | | | |
| CHK1-ECOR1-F | 5'-GGATGAATTCATGGCAGTGCCTTTTGTG-3' | This study | Cloning of CHK1 into pCS2-GFP Zebrafish vector |
| CHK1-XBA1-R | 5'-GAGATCTAGATGTGGCAGGAAGCCAA-3' | | |
| **Primers for site-directed mutagenesis** | | | |
| CHK1-S345A-F | 5'-GTGGGCTGGGCAAAGCTGATCCCTTGTACC-3' | This study | Generation of CHK1-S345A phosphorylation mutant |
| CHK1-S345A-R | 5'-GGTACAAGGGATCAGCTTTGCCCAGCCCAC-3' | | |
| CHK1-S317A-F | 5'-GGGGTTCTGGCTGAGCACTGGAGTACTTCA-3' | This study | Generation of CHK1-S317A phosphorylation mutant |
| CHK1-S317A-R | 5'-GTGAAGTACTCCAGTGCTCAGCCAGAACCC-3' | | |
| CHK1-D130A-F | 5'-CAGAAGATTTTCTGGTTTAATAGCCCTGTGAGTTATTCCAATACC-3' | This study | Generation of CHK1-D130A catalytic mutant |
| CHK1-D130A-R | 5'-GGTATTGGAATAACTCACAGGGCTATTAAACCAGAAAATCTTCTG-3' | | |
| CHK1-237stopF | 5'-TTGGAAAAAAATCGATTCTTAACCTCTAGCTCTGCTGCA-3' | This study | Generation of CHK1-CP1 fragment |
| CHK1-237stopR | 5'-TGCAGCAGAGCTAGAGGTTAAGAATCGATTTTTTTCCAA-3' | | |
| CHK1-293stopF | 5'-ATTTTCTAAGTAGATTCAATCCAATTTGGACTTCTCTCCA-3' | This study | Generation of CHK1-CP2 fragment |
| CHK1-293stopR | 5'-TGGAGAGAAGTCCAAATTGGATTGAATCTACTTAGAAAAT-3' | | |
| CHK1-338stopF | 5'-ACATTGATAAATAGGTACAAGGGATCAGCTTTT-3' | This study | Generation of CHK1-CP3 fragment |

| | | | |
|---|---|---|---|
| CHK1-338stopR | 5'-AAAAGCTGATCCCTTGTACCTATTTATCAATGT-3' | | |
| SPRTN-S373A-F | 5'-CTAGTTCTCAGAGAAGGGTTGCATCTTCTAAGATATCCCTAAG-3' | This study | Generation of SPRTN-S373A phosphodefficient mutant |
| SPRTN-S373A-R | 5'-CTTAGGGATATCTTAGAAGATGCAACCCTTCTCTGAGAACTAG-3' | | |
| SPRTN-S374A-F | 5'-GTTCTCAGAGAAGGGTTTCAGCTTCTAAGATATCCCTAAGAAG-3' | This study | Generation of SPRTN-S374A phosphodefficient mutant |
| SPRTN-S374A-R | 5'-CTTCTTAGGGATATCTTAGAAGCTGAAACCCTTCTCTGAGAAC-3' | | |
| SPRTN-S383A-F | 5'-CTAAGATATCCCTAAGAAATTCTGCAAAAGTAACGGAATCAGCATCT-3' | This study | Generation of SPRTN-S383A phosphodefficient mutant |
| SPRTN-S383A-R | 5'-AGATGCTGATTCCGTTACTTTTGCAGAATTTCTTAGGGATATCTTAG-3' | | |
| SPRTN-S373A,S374A-F | 5'-CTTCTAGTTCTCAGAGAAGGGTTGCAGCTTCTAAGATATCCCTAAGAAAT-3' | This study | Generation of SPRTN-S373A,S374A double phosphomimetic mutant |
| SPRTN-S373A,S374A-R | 5'-ATTTCTTAGGGATATCTTAGAAGCTGCAACCCTTCTCTGAGAACTAGAAG-3' | | |
| SPRTN-S373E-F | 5'-GTCTCTTCTAGTTCTCAGAGAAGGGTTGAGTCTTCTAAGATATCCCTAAGAAATTC-3' | This study | Generation of SPRTN-S373E phosphomimetic mutant |
| SPRTN-S373E-R | 5'-GAATTTCTTAGGGATATCTTAGAAGACTCAACCCTTCTCTGAGAACTAGAAGAGAC-3' | | |
| SPRTN-S374E-F | 5'-CTTCTAGTTCTCAGAGAAGGGTTTCAGAGTCTAAGATATCCCTAAGAAATTCT-3' | This study | Generation of SPRTN-S374E phosphomimetic mutant |
| SPRTN-S374E-R | 5'-AGAATTTCTTAGGGATATCTTAGACTCTGAAACCCTTCTCTGAGAACTAGAAG-3' | | |
| SPRTN-S383E-F | 5'-CTTCTAAGATATCCCTAAGAAATTCTGAGAAAGTAACGGAATCAGCATCTGTGATG-3' | This study | Generation of SPRTN-S383E phosphomimetic mutant |
| SPRTN-S383E-R | 5'-CATCACAGATGCTGATTCCGTTACTTTCTCAGAATTTCTTAGGGATATCTTAGAAG-3' | | |
| **dsDNA probe for SPRTN cleavage assay** | | | |
| dsDNA-100bp | 5'-CGCGGGTTAGCGGTACCCAGTCCAGTGACCTAGGCAGCTTTAAGCTAGTACGACTTGCTTAGATTGCAGTCGACGACGTAGCTGGCATAGAGGTACAGC-3' | | |
