# Supplementary material for: SPRTN protease and checkpoint kinase 1 cross-activation loop safeguards DNA replication
Source: Nat Commun. 2019 Jul 17;10:3142. doi: 10.1038/s41467-019-11095-y (PMC6637133; doi:10.1038/s41467-019-11095-y)
Supplement: Supplementary file 4 — Description of Additional Supplementary Data 1 [file 41467_2019_11095_MOESM4_ESM.pdf]

Description of Additional Supplementary Files

File Name: Supplementary Data 1
Description: Mass Spectrometry data for phosphosite search.
