# Supplementary material for: SPRTN protease and checkpoint kinase 1 cross-activation loop safeguards DNA replication
Source: Nat Commun. 2019 Jul 17;10:3142. doi: 10.1038/s41467-019-11095-y (PMC6637133; doi:10.1038/s41467-019-11095-y)
Supplement: Supplementary file 5 — Reporting Summary [file 41467_2019_11095_MOESM5_ESM.pdf]

nature research

Corresponding author(s): K.Ramadan NCOMS-19-14005A

Reporting Summary

Nature Research wishes to improve the reproducibility of the work that we publish. This form provides structure for consistency and transparency in reporting. For further information on Nature Research policies, see Authors & Referees and the Editorial Policy Checklist.

Statistical parameters

When statistical analyses are reported, confirm that the following items are present in the relevant location (e.g. figure legend, table legend, main text, or Methods section).

n/a	Confirmed	
☐	☒	The exact sample size (*n*) for each experimental group/condition, given as a discrete number and unit of measurement
☐	☒	An indication of whether measurements were taken from distinct samples or whether the same sample was measured repeatedly
☐	☒	The statistical test(s) used AND whether they are one- or two-sided<br>*Only common tests should be described solely by name; describe more complex techniques in the Methods section.*
☒	☐	A description of all covariates tested
☒	☐	A description of any assumptions or corrections, such as tests of normality and adjustment for multiple comparisons
☐	☒	A full description of the statistics including central tendency (e.g. means) or other basic estimates (e.g. regression coefficient) AND variation (e.g. standard deviation) or associated estimates of uncertainty (e.g. confidence intervals)
☐	☒	For null hypothesis testing, the test statistic (e.g. *F*, *t*, *r*) with confidence intervals, effect sizes, degrees of freedom and *P* value noted<br>*Give P values as exact values whenever suitable.*
☒	☐	For Bayesian analysis, information on the choice of priors and Markov chain Monte Carlo settings
☒	☐	For hierarchical and complex designs, identification of the appropriate level for tests and full reporting of outcomes
☒	☐	Estimates of effect sizes (e.g. Cohen's *d*, Pearson's *r*), indicating how they were calculated
☐	☒	Clearly defined error bars<br>*State explicitly what error bars represent (e.g. SD, SE, CI)*

Our web collection on statistics for biologists may be useful.

Software and code

Policy information about availability of computer code

Data collection	ImageLab (BioRad) v5.2.1<br>ImageJ v1.46r
Data analysis	GraphPad Prism v6.01<br>PEAKS v7.5<br>Xcalibur Qual Browser<br>FlowJo

For manuscripts utilizing custom algorithms or software that are central to the research but not yet described in published literature, software must be made available to editors/reviewers upon request. We strongly encourage code deposition in a community repository (e.g. GitHub). See the Nature Research guidelines for submitting code & software for further information.

Data

Policy information about availability of data

All manuscripts must include a data availability statement. This statement should provide the following information, where applicable:

- Accession codes, unique identifiers, or web links for publicly available datasets
- A list of figures that have associated raw data
- A description of any restrictions on data availability

The Mass Spectrometry raw data reported in this paper have been deposited in the ProteomeXchange Consortium via the PRIDE59 partner repository with the dataset identifier PXD006741.
All other data supporting the findings are available from the corresponding author at a reasonable request.

Field-specific reporting

Please select the best fit for your research. If you are not sure, read the appropriate sections before making your selection.

☒ Life sciences ☐ Behavioural & social sciences ☐ Ecological, evolutionary & environmental sciences

For a reference copy of the document with all sections, see nature.com/authors/policies/ReportingSummary-flat.pdf

Life sciences study design

All studies must disclose on these points even when the disclosure is negative.

Sample size	Most experiments were reproduced at least three times with similar results. Experiments repeated twice were those involving zebrafish and in figures 2c, 4d, 6j, 8c and S3b. For zebrafish experiments, between 6-28 individuals were used per condition and experiment. For DNA fibers, more than 100 were analysed per condition per experiment.
Data exclusions	No relevant data was excluded from this study.
Replication	The vast majority of experiments were reproduced at least three times with similar results. Experiments repeated twice were those involving zebrafish and in figures 2c, 4d, 6j, 8c and S3b .
Randomization	In each experiment, different cell samples started from similar conditions and treatments were randomly allocated.
Blinding	The experiment in Figure 7a-c was carried out blindly. The investigator performing the analysis of DNA fibers did not know what protein was overexpressed in each cell sample.

Reporting for specific materials, systems and methods

Materials & experimental systems

n/a	Involved in the study	
☐	☒	Unique biological materials
☐	☒	Antibodies
☐	☒	Eukaryotic cell lines
☒	☐	Palaeontology
☐	☒	Animals and other organisms
☒	☐	Human research participants

Methods

n/a	Involved in the study	
☒	☐	ChIP-seq
☐	☒	Flow cytometry
☒	☐	MRI-based neuroimaging

Unique biological materials

Policy information about availability of materials

Obtaining unique materials	All unique materials used in this study are readily available from the authors

Antibodies

Antibodies used	A full description of all antibodies used in this study is provided in Supplementary Table 2: "Supplementary Information"
Validation	In this study, we have validated antibody CST #6950 (RRID:AB_10827652) to recognize CHK1 target sequences. It is able to detect phosphorylated CHK1 target sequences on substrates as a result of CHK1 activity in in vitro phosphorylation assays, but not when CHK1 activity is chemically inhibited by the CHK1 inhibitor UCN-01.<br>For all other antibodies, validation was done by companies.

Eukaryotic cell lines

Policy information about cell lines

Cell line source(s)	A full description of all cell lines used in this study is provided in Supplementary Table 2: "Supplementary Information".
Authentication	U2OS, HeLa, HEK293 and T24 cells used in this study were originally obtained from ATCC and no further genetically authenticated.
Mycoplasma contamination	All cell lines used in this study were tested negative to mycoplasma by the MycoAlertTM Mycoplasma Detection Kit (Lonza LT07-218).
Commonly misidentified lines<br>(See ICLAC register)	No misidentified cell lines were used in this study. HEK293 cells morphology was uniform and different from HeLa cells.

Animals and other organisms

Policy information about studies involving animals; ARRIVE guidelines recommended for reporting animal research

Laboratory animals	A description of zebrafish animal models used in this study is provided in Supplementary Table 2: "Supplementary Information".
Wild animals	This study did not involve wild animals.
Field-collected samples	This study did not involve samples collected from the field.

Flow Cytometry

Plots

Confirm that:

☒ The axis labels state the marker and fluorochrome used (e.g. CD4-FITC).

☒ The axis scales are clearly visible. Include numbers along axes only for bottom left plot of group (a 'group' is an analysis of identical markers).

☒ All plots are contour plots with outliers or pseudocolor plots.

☒ A numerical value for number of cells or percentage (with statistics) is provided.

Methodology

Sample preparation	For cell cycle analysis of cultured HEK293 cells, SPRTN was depleted by siRNA for three days, or CHK1 was overexpressed for two days. Cells were incubated with 10 µM EdU for 30 minutes and after trypsinization, 200,000 cells were washed and fixed in 4% formaldehyde. Following Click-iT reaction with EdU Alexa Fluor® 647 imaging kit (ThermoFisher Scientific), cells were then stained using phospho-Histone H3 (S10) Alexa Fluor® 488 conjugate (Cell Signalling), and Propidium Iodide.
Instrument	FACScalibur (BD Biosciences)
Software	Data was analyzed with FlowJo.
Cell population abundance	The percentage of cells in each cell cycle phase was assessed and plotted
Gating strategy	Only isolated alive cells were analyzed.

☒ Tick this box to confirm that a figure exemplifying the gating strategy is provided in the Supplementary Information.
