# Supplementary material for: SPRTN protease and checkpoint kinase 1 cross-activation loop safeguards DNA replication
Source: Nat Commun. 2019 Jul 17;10:3142. doi: 10.1038/s41467-019-11095-y (PMC6637133; doi:10.1038/s41467-019-11095-y)
Supplement: Supplementary file 2 — Peer Review File [file 41467_2019_11095_MOESM2_ESM.pdf]

Editorial Note: This manuscript has been previously reviewed at another journal that is not operating a transparent peer review scheme. The manuscript was considered suitable for publication without further review at *Nature Communications*.
